# Electrochemically Enhanced Low-Impedance Ti_3_C_2_T_*x*_ MXene Epidermal Electrodes for Accurate Electrophysiological Monitoring

**DOI:** 10.1007/s40820-026-02132-9

**Published:** 2026-03-20

**Authors:** Liubing Fan, Fangfang Gao, Liangxu Xu, Xiaochen Xun, Shuchang Zhao, Bing Yang, Han Bi, Xuan Zhao, Qingliang Liao, Yue Zhang

**Affiliations:** 1 Academy for Advanced Interdisciplinary Science and Technology, Beijing Advanced Innovation Center for Materials Genome Engineering, University of Science and Technology Beijing, Beijing, 100083 People’s Republic of China; 2 Key Laboratory of Advanced Materials and Devices for Post-Moore Chips, Ministry of Education, Beijing Key Laboratory for Advanced Energy Materials and Technologies, School of Materials Science and Engineering, University of Science and Technology Beijing, Beijing, 100083 People’s Republic of China

**Keywords:** Two-dimensional materials, MXene, Epidermal electrodes, Electrophysiology, Wearable healthcare

## Abstract

Accurate monitoring of electrophysiological signals through epidermal electrodes is crucial for advancing human–machine interfaces and wearable healthcare. While highly conductive materials are conventionally used as epidermal electrodes, their limited electrochemical performance results in high interfacial impedance and consequent signal distortion. Here, we present an electrochemically enhanced low-impedance Ti_3_C_2_T_*x*_ MXene epidermal electrode for accurate electrophysiological monitoring. The low interfacial impedance is achieved by producing and bridging large Ti_3_C_2_T_*x*_ MXene nanosheets. Large MXene nanosheets were prepared by combining precursor particle sedimentation with mild shear-assisted exfoliation. An orderly stacking structure was constructed through hydroxyethyl cellulose (HEC) crosslinking large MXene nanosheets to enhance electrochemical performance and flexibility. The epidermal electrodes were fabricated by bonding HEC/MXene film to poly(dimethylsiloxane) substrate via *in*-*situ* curing. The MXene epidermal electrodes exhibit lower interfacial impedance (53 kΩ cm^2^ at 10 Hz) compared to standard Ag/AgCl gel electrodes (436 kΩ cm^2^ at 10 Hz). This reduction results in a 2.4-fold improvement in signal-to-noise ratio, enabling accurate electrophysiological monitoring. A miniature recording system is integrated with the epidermal electrodes to monitor electrophysiological signals in wearable scenes. Physiological applications have been validated in gesture recognition and health monitoring. Therefore, the electrochemically enhanced low-impedance MXene epidermal electrodes offer a reliable option for acquiring high-fidelity electrophysiological signals.

**Supplementary Information:**

The online version contains supplementary material available at 10.1007/s40820-026-02132-9.

## Highlights

- Large Ti_3_C_2_T_*x*_ MXene (~6.5 μm) was prepared through combined precursor particle sedimentation and mild shear-force-assisted delamination to enhance conductivity and capacitance.
- The epidermal electrode with orderly stacking structure was constructed using hydrogen bonding crosslinking and *in-situ* curing, demonstrating lower interfacial impedance (53 kΩ cm^2^ at 10 Hz) and higher signal-to-noise (39 dB) compared to commercial Ag/AgCl gel electrode.
- The portable recording system was integrated with the epidermal electrodes, enabling applications in muscular status and healthcare monitoring.

**Supplementary Information:**

The online version contains supplementary material available at 10.1007/s40820-026-02132-9.

## Graphical abstract

**Figure:**
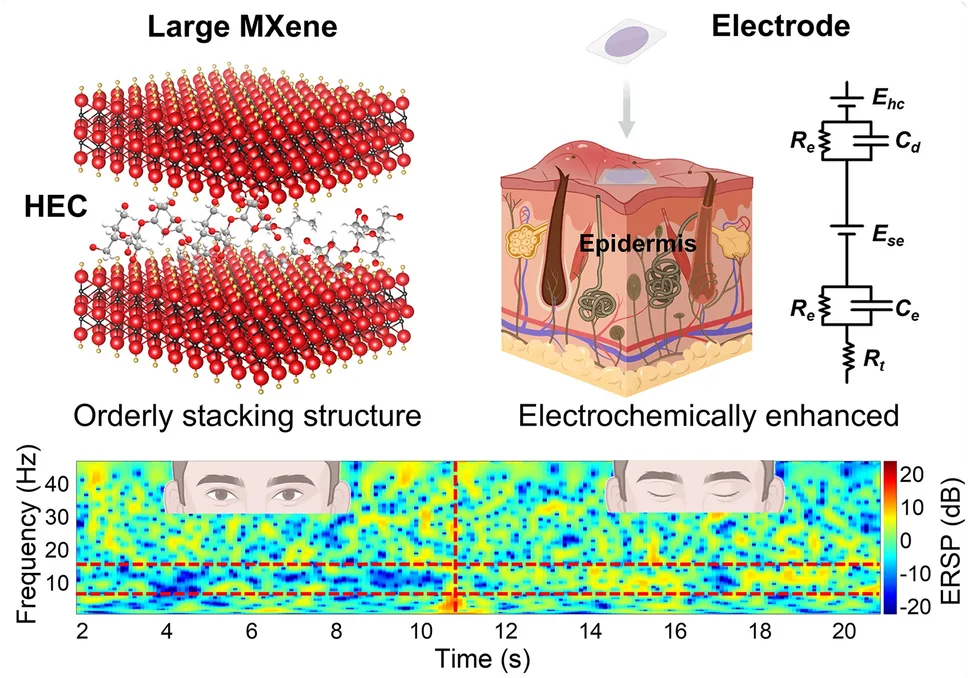

## Introduction

Epidermal bioelectronics attached to the skin and capable of acquiring electrophysiological signals represent a promising avenue for athletic training [1], human–machine interfaces (HMIs) [2], and wearable healthcare [3]. High-fidelity signal acquisition is crucial for enhancing diagnostic accuracy in medicine and optimizing the performance of HMIs [4, 5]. The generation of these signals is rooted in biopotentials, which are transmitted across the electrode–skin interface and give rise to an electron-ionic layer [6, 7]. Therefore, accurate monitoring of electrophysiological signals requires epidermal electrodes with low interfacial impedance, which necessitates both conductivity and electrochemical activity.

The epidermal electrode materials can primarily be categorized into metal films [8], conductive polymers [9], and nano-carbon materials [2]. Although metal films offer high conductivity, they possess limited electrochemical activity and relatively high cost [10]. Conductive polymers are known for excellent flexibility, but their lower conductivity restricts practical application [11]. Nano-carbon materials, such as graphene and carbon nanotubes, have been successfully applied in various flexible wearable sensors [12]. However, they still face specific challenges when employed as high-fidelity, low-impedance epidermal electrodes with conformal skin contact. For instance, electrodes based on these materials often require a trade-off among conductivity, electrochemical activity, and mechanical properties [13]. Consequently, the development of low-impedance epidermal electrodes remains a material challenge. In the current study, Rogers et al. fabricated a filamentary serpentine Au electrode, which achieved enhanced stretchability for use in epidermal electronics [14]. Lu et al. developed a graphene-based tattoo electrode that shows excellent conformability and reliable acquisition of various electrophysiological signals [15]. Ouyang et al. modified PEDOT:PSS to produce an epidermal electrode with excellent adhesion properties [9]. Although these advances have improved device flexibility and skin compatibility, most prior efforts have predominantly focused on lowering impedance through geometric or mechanical design of the electrodes, while the optimization of the intrinsic electrochemical properties in electrode materials has received comparatively limited attention [16, 17].

MXenes, a family of two-dimensional (2D) transition metal carbides, nitrides, and carbonitrides, have attracted great attention due to their unique properties and diverse applications [18]. Currently, the synthesis methods for MXenes have become diverse and versatile, including wet chemical, electrochemical, molten salt, and chemical vapor deposition [19]. These materials feature abundant surface terminal groups, such as hydroxyl groups (-OH), oxygen (-O), and fluorine (-F), that facilitate surface functionalization and enable the formation of composites with other materials for the development of hydrogels, aerogels [20, 21]. For instance, Cai et al. developed an MXene-based electronic skin (e-skin) system featuring a heterostructure that incorporates an MXene layer. This e-skin exhibited an exceptional working range (2800%) and was capable of monitoring multi-dimensional tensile motions, tactile pressure, and various other signals [22]. These MXene-based composites demonstrate promising application prospects in intelligent sensing and flexible electronics [23]. Meanwhile, MXenes possess metallic conductivity and distinctive electrochemical properties. These inherent properties not only underpin their success in energy storage devices but also make them attractive for wearable electronic interfaces [24]. Leveraging these characteristics, researchers have developed several MXene epidermal electrodes for electrophysiological monitoring [25, 26]. A bioelectronic interface termed “MXtrodes” was fabricated by combining MXene ink with a polymer substrate, enabling high resolution and large-scale electrophysiological signal acquisition with performance superior to that of conventional materials [25]. An e-skin based on an MXene/CNT and thermoplastic polyurethane (TPU) textile scaffold was developed for the acquisition of multiple biopotentials [27]. Therefore, MXene is an appropriate candidate for fabricating epidermal electrodes. However, precise tuning of MXene flake sizes and electrochemical properties has received comparatively less attention [28, 29]. Extensive research has demonstrated that large lateral dimensions of MXene can enhance performance [30]. Nevertheless, conventional ultrasonication-assisted delamination severely compromises the size of MXene flakes. Although hand-assisted soft delamination can produce larger MXene sheets (up to 40 μm), its efficiency is relatively low [31].

In this work, we have successfully fabricated an electrochemically enhanced low-impedance Ti_3_C_2_T_*x*_ MXene epidermal electrode for accurate electrophysiological signal monitoring. A mild shear-force-assisted delamination method was developed to prepare large Ti_3_C_2_T_*x*_ MXene flakes. An orderly stacking structure was constructed through hydrogen bonding crosslinking, which enhanced the electrochemical performance of the material. The *in*-*situ* curing process ensured conformal contact between the electrode and the skin. The large Ti_3_C_2_T_*x*_ MXene nanosheets with an average lateral size of 6.5 μm were synthesized via particle sedimentation treatment of MAX phase, followed by mild shear-force-assisted delamination. The large MXene exhibits enhancement in conductivity and capacitance compared to MXene prepared via conventional sonication methods. The orderly stacking structure was constructed through hydroxyethyl cellulose (HEC) interfacial bridging large MXene nanosheets to improve electrochemical performance and flexibility. A patterned freestanding HEC/MXene film was bonded with poly(dimethylsiloxane) (PDMS) through *in*-*situ* curing. The thin and tough PDMS substrate enables the epidermal electrode to adhere conformally to the skin surface. The HEC/MXene epidermal electrodes exhibit excellent capacitance with high conductivity, which collectively reduces interfacial impedance. Compared to commercial Ag/AgCl gel electrodes, the prepared epidermal electrodes achieve a reduced interfacial impedance with a higher signal-to-noise ratio (SNR) in electrophysiological signal monitoring. We integrated a miniature recording system with the electrodes to acquire electrophysiological signals in wearable scenes. We demonstrate the applications of HEC/MXene epidermal electrodes in gesture recognition and wearable healthcare. Our electrochemically enhanced low-impedance MXene epidermal electrodes provide a reliable candidate for accurate electrophysiological monitoring.

## Experimental Section

### Materials

The pristine Ti_3_AlC_2_ MAX phase (200 mesh) was purchased from Jilin 11 Technology Co., Ltd. Lithium fluoride (LiF, 99.99%) was purchased from Shanghai Boer Chemical Reagents Co., Ltd. Hydrochloric acid (HCl, 36.0% ~ 38.0%) was supplied by Sinopharm Chemical Reagent Co., Ltd. Hydroxyethyl Cellulose (HEC, C_29_H_52_O_21_, 1000 ~ 1500 mPa s, 25 °C) was obtained from Shanghai Aladdin Biochemical Technology Co., Ltd. Phosphate buffered saline (PBS, 0.01 M, pH 7.4) and artificial sweat (pH 6.5) were purchased from Shanghai Macklin Biochemical Co., Ltd. Poly(dimethylsiloxane) (PDMS, Sylgard 184) was from Dow Inc.

### Preparation of Ti_3_C_2_T_x_ MXene Nanosheets with Various Flake Sizes

For large MAX phase preparation, the separation principle based on Eq. (1):

1 $$v = \frac{{g(\rho_{p} - \rho_{l} )d^{2} }}{18u}$$

where *v* is the terminal velocity (m s^−1^), *g* is the gravitational acceleration (m s^−2^), *ρ*_*p*_ is the mass density of Ti_3_AlC_2_ MAX phase (~ 4.2 × 10^3^ kg m^−3^), *ρ*_*l*_ is the mass density of water (1.0 × 10^3^ kg m^−3^), *d* is the diameter of MAX phase, and *u* is the dynamic viscosity (Pa) of water (8.9 × 10^–4^ Pa at ~ 25 °C). Large MAX powder has a higher settling velocity, causing it to deposit preferentially at the bottom of the sediment and thereby remove small powder from the supernatant [30]. The large MAX sample was prepared by dispersing pristine Ti_3_AlC_2_ MAX phase (200 mesh) in a water column 17.5 cm in height, followed by magnetic stirring for 10 min and subsequent settling under static conditions for 7 min. The supernatant was decanted from the sediment, and this process was repeated three times as described. Finally, the resulting sediment was vacuum-dried at 60 °C for 10 h. The final product was designated as processed MAX to distinguish it from the pristine MAX. Ti_3_C_2_T_*x*_ MXene nanosheets with varying flake sizes were synthesized by selectively etching the Al layer from Ti_3_AlC_2_ MAX phase in an HCl and LiF solution [32]. For the synthesis of small Ti_3_C_2_T_*x*_ MXene nanosheets, LiF powder (1.6 g) was dissolved in 9 M HCl (20 mL) and magnetically stirred for 15 min in a Teflon vessel. Subsequently, pristine Ti_3_AlC_2_ MAX powder (1.0 g) was slowly added, and the mixture was stirred magnetically at 50 °C for 48 h. The resulting product was washed 6–7 times with deionized water (DI) via iterative centrifugation at 3500 rpm for 5 min until the supernatant reached a pH of approximately 6. The resultant swollen sediment was re-dispersed in DI water and subjected to sonication under an argon atmosphere in an ice bath for 30 min (300 W). After centrifugation at 3500 rpm for 30 min, the supernatant containing small Ti_3_C_2_T_*x*_ MXene nanosheets was collected. For medium Ti_3_C_2_T_*x*_ nanosheets, the etching procedure was identical to that used for small nanosheets, but the exfoliation step was modified. The medium nanosheets were obtained by applying vortex oscillation to the dispersion at 2000 rpm for 30 min, followed by centrifugation at 3500 rpm for 30 min. Large MXene nanosheets were synthesized using processed Ti_3_AlC_2_ MAX powder as the precursor, with all other experimental conditions kept the same as those for the medium MXene.

### Fabrication of HEC/MXene Epidermal Electrodes

The HEC solution was prepared by dissolving HEC powder in DI water at a concentration of 5 mg mL^−1^. The HEC solution was added to a freshly prepared large MXene dispersion (12.9 mg mL^−1^) at HEC weight fractions of 0, 5, 10, 15, and 20 wt%. Small, medium, and large MXene films, as well as HEC/MXene films, were prepared via vacuum-assisted filtration. The obtained freestanding films were cut into circular shapes using a laser engraving machine (LaserMen, LM-6040, power 0.4 W). Considering the wearability and portability of the electrode, the diameter was set to 10 mm, corresponding to an actual usable area of 0.785 cm^2^. A volume of 1 mL PDMS (base to curing agent ratio of 10:1) was spin-coated on a polyethylene terephthalate (PET) substrate at 800 rpm for 30 s. The PET was fixed onto a glass slide to ensure a flat surface. The patterned freestanding MXene films were placed vertically on the uncured PDMS layer and then vacuum-dried at 90 °C for 2 h. The epidermal electrodes were obtained by peeling off the electrode from the PET substrate. The resulting PDMS layer had a thickness of approximately 0.1 mm.

### Characterization

Scanning electron microscopy (SEM) (FEI Quanta 3D FEG) was used to characterize the morphology and microstructure of MXene nanosheets and films at an acceleration voltage of 10 kV. SEM samples were prepared by drop casting MXene dispersions onto porous alumina membrane (0.2 μm pore size, Anodisc, Whatman). Transmission electron microscopy (TEM) (JEOL JEM-2010) was used to characterize the morphology of MXene nanosheets at 200 kV. TEM samples were prepared by deposition of MXene nanosheets onto a 300-mesh copper grid. To analyze the cross-sectional morphology of the electrodes, the samples were prepared via focused-ion beam (FIB) (Thermo Fisher Scientific Scios 2 DualBeam) technique. High-resolution TEM/STEM characterizations were then carried out using spherical aberration (Cs) corrected FEI Themis Z at 300 kV. SEM images were analyzed using the Nano Measurer software to calculate the lateral dimensions of medium and large MXene nanosheets. The lateral size refers to the length (*x*, the longest distance) of the MXene nanosheet, while the width (*y*) is the shortest distance of the nanosheet. Dynamic light scattering (DLS) was characterized using a Zetasizer (Malvern Instruments, USA, Nano ZS90) to determine the dimensions of small MXene nanosheets. The dimensions were taken from the average of five measurements. Atomic force microscopy (AFM) (Bruker Dimension Icon system) was employed to analyze the topography of MXene nanosheets using ScanAsyst atomic force microscopy scan mode. AFM samples were prepared by drop casting MXene dispersions on silicon wafers. X-ray diffraction (XRD) measurement was characterized by the Rigaku SmartLab (9 kW) X-ray diffractometer using Cu- Kα radiation with a step scan of 0.013°. Wide-angle X-ray scattering (WAXS) was characterized by Xenocs Xeuss 3.0, which used a Cu-Kα X-ray beam (8 keV) and an Eiger2R 1M detector (1028 × 1042 pixels). The samples were 5-mm-wide, 10-mm-long strips. The X-ray beam was parallel to the film plane and struck the film cross-section. The sample-detector distance was 60 mm. Herman’s orientation factor (*f*) was calculated to quantify the alignment degree of MXene flakes relative to the film plane using Eq. (2):

2 $$f = \frac{{3\langle \cos^{2} \phi \rangle - 1}}{2}$$

where $\left\langle {\cos^{2} \phi } \right\rangle$ is the mean-square cosine of the azimuthal angle for the (002) peak of the MXene film, which is calculated from the scattered intensity at an azimuthal angle *ϕ* according to Eq. (3):

3 $$\left\langle {\cos^{2} \phi } \right\rangle = \frac{{\int_{0}^{{\frac{\pi }{2}}} I (\phi )\sin \phi \cos^{2} \phi {\mkern 1mu} d\phi }}{{\int_{0}^{{\frac{\pi }{2}}} I (\phi )\sin \phi {\mkern 1mu} d\phi }}$$

*f* = 1 represents all MXene flakes parallel to the film plane, and *f* = 0 shows the random alignment of MXene flakes. UV–vis absorption spectra were recorded using a Shimadzu UV-3600i Plus spectrophotometer over a wavelength range of 200 to 1000 nm. The MXene dispersion was diluted to a concentration of 0.007 mg mL^−1^ before measurement. All spectra were normalized to the absorbance intensity at 220 nm, and the intensity at 260 nm was extracted as an index of MXene nanosheet concentration. Fourier transform infrared (FTIR) spectroscopy was performed in attenuated total reflectance (ATR) mode using a Thermo Fisher Scientific Nicolet IS50 spectrometer. Raman spectrum acquired with a Horiba LabRam Odyssey with a 532 nm laser at 25% laser power. X-ray photoelectron spectroscopy (XPS) measurements were conducted on a Nexsa G2 (Thermo Fisher Scientific) equipped with a monochromatic Al-Kα X-ray source operated at 120 W, with a step size of 1 eV. All electrochemical measurements were tested using an Autolab PGSTAT204 (Metrohm, Switzerland) electrochemical workstation. Cyclic voltammetry (CV) measurements were performed with 1 × PBS and artificial sweat in a 3-electrode configuration with an Ag/AgCl reference electrode and a graphite rod counter electrode. The scan rate of CV was usually 20 mV/s^–1^, and in double-layer capacitor tests, it ranged from 1 to 6 mV s_–1_. Current pulsing was performed using chronopotentiometry. Electrical conductivity was measured using a four-point probe technique (Keithley 2400) with a uniform probe spacing of 1.0 mm. Electrode–skin interfacial impedance was recorded using a three-electrode system, with the distance between electrodes set at approximately 3.0 cm. The skin of subjects was cleaned with 75% alcohol wipe before interfacial impedance measurements. Adhesion force was measured using a universal tensile tester (Mark-10, ESM303 system).

### Electrophysiological Measurements

All electrophysiological signals were recorded using a g.Hiamp research amplifier (G.tec, Austria) at a sampling rate of 19,200 Hz and a notch filter (48 to 52 Hz). The portable recording system for EMG signals utilizes an INA128 amplifier with an input impedance of 10 GΩ, a CMRR of 120 dB, and a sampling rate of 500 Hz. The skin of subjects was cleaned with 75% alcohol wipe before measurements. Commercial Ag/AgCl gel electrodes from Cathay Manufacturing Corp. (CH4349TD, gel diameter ≈ 16 mm, actual usable area ≈ 2.011 cm^2^) were used as the control. The change rates of mean power frequency (MPF) were calculated based on the values recorded in the respective zones. MPF_s_ refers to the value during the initial 10-s interval, while MPF_e_ indicates the value at the final 10-s interval. ΔMPF is defined as the difference between MPF_s_ and MPF_e_. All EMG data were digitally filtered with a bandpass filter set to 5–250 Hz. The RMS envelope of the filtered signal was computed with a 200 ms moving window with a 25 ms overlap. A trial was defined as a single muscle contraction. For each trial, the signal was defined as the maximum value of the RMS envelope during contraction (max RMS_Contraction_), whereas noise was defined as the average value of the RMS envelope in the 1 s preceding the contraction (baseline region) (mean RMS_Baseline_). The SNR_*n*_ in each trial was calculated using these two values, according to Eq. (4):

4 $${\mathrm{SNR}}_{{\mathrm{n}}} = 20\log_{10} \left( {\frac{{\max {\mathrm{RMS}}_{{{\mathrm{Contraction}}}} }}{{{\mathrm{meanRMS}}_{{{\mathrm{Baseline}}}} }}} \right)$$

The overall SNR was the average of SNR_*n*_ with *n* = 8 trials. Multi-cycle ECG monitoring was performed at 20-min intervals. All ECG data were digitally filtered with a bandpass filter (1 to 50 Hz). For EOG monitoring, two epidermal electrodes were attached laterally to the right and left outer canthi to record horizontal EOG. Two epidermal electrodes were positioned above and below the eye to record vertical EOG in another channel, with a reference electrode attached behind the ear. All EOG data were digitally filtered with a bandpass filter (2 to 60 Hz). For EEG recording, two epidermal electrodes were positioned at Fp1 and Fp2 according to the international 10–20 electrode placement system. All EEG data were digitally filtered using a bandpass filter (0.5 to 50 Hz).

### In Vitro Biocompatibility Test

The Human skin fibroblast (HSF) cell line was used in this study (supplied by Cellverse Co., Ltd.; Research Resource Identifier (RRID): CVCL_C9G0). HEC/MXene films were sterilized under UV light for 60 min. HSF cells were cultured in an incubator at 37 °C with 5% CO_2_. The films were placed in 24-well plates, and 1000 μL of cell suspension (density 6 × 10^4^ cells mL^−1^) was seeded on them. Cell viability was determined using the CCK-8 kit. Absorbance was measured at 450 nm using a microplate reader. The HSF cells were stained with a live/dead kit: Calcein-AM (live cells, green) and PI (dead cells, red). Fluorescent images were captured using a fluorescence microscope.

### Ethics Oversight

All human experiments in this study were approved by the Human Research Ethics Review Committee for the University of Science and Technology Beijing (approval number: 2025–4-106). All participants provided written informed consent.

## Results and Discussion

### Design Principle of Low-Impedance Epidermal Electrodes

**Fig. 1:**
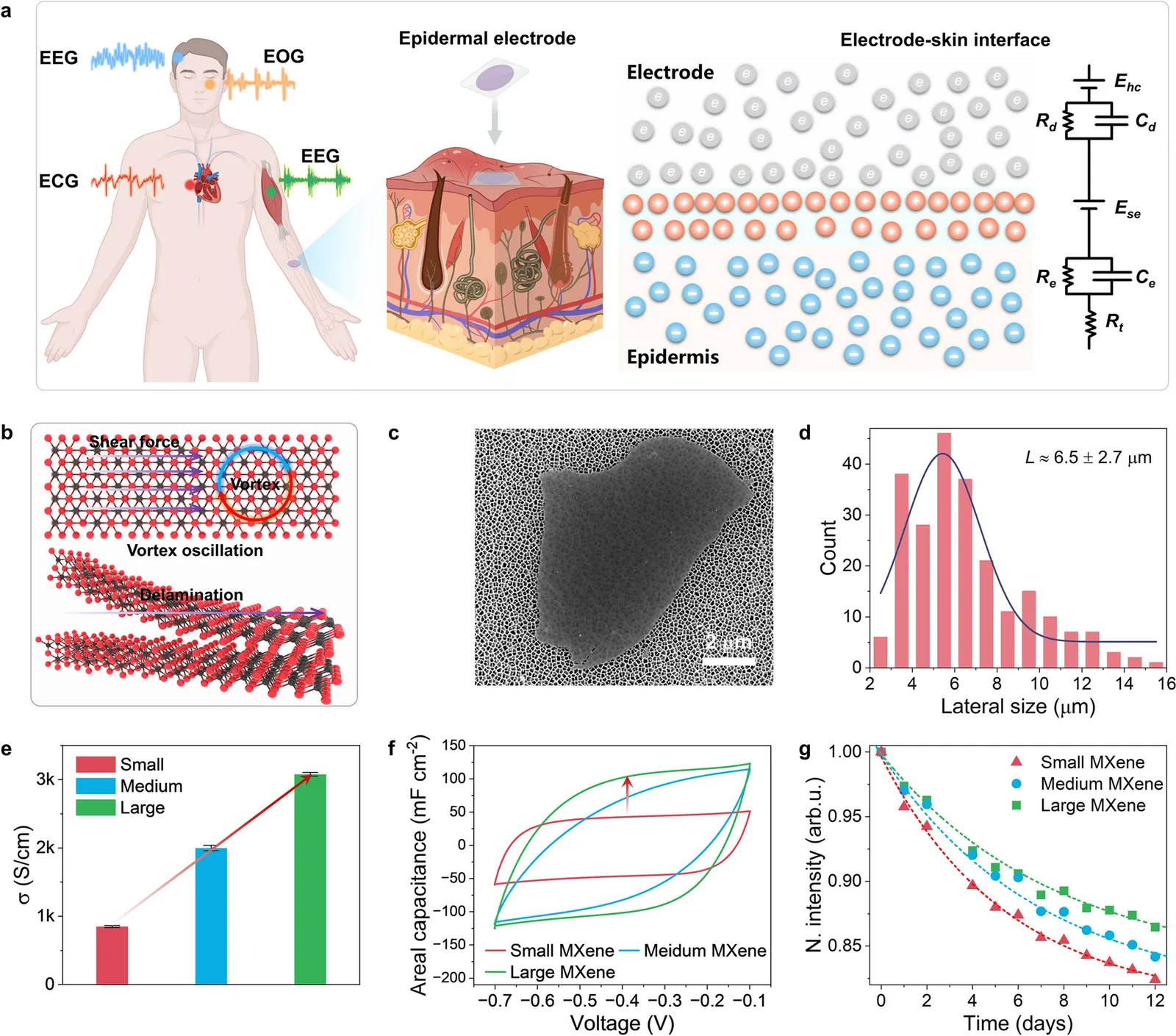
Materials design rationale and characterization of Ti_3_C_2_T_*x*_ MXene nanosheets for epidermal electrodes. **a** Schematic illustration of electrophysiological signals and the electrode–skin interface with an equivalent circuit model. **b** Schematic illustration of the delamination process for synthesizing large Ti_3_C_2_T_*x*_ MXene nanosheets via vortex oscillation. **c** SEM image of the as-synthesized large Ti_3_C_2_T_*x*_ MXene. **d** Statistical distribution of the lateral flake size of large Ti_3_C_2_T_*x*_ MXene using SEM images. Data are presented as mean ± SD. **e** Comparison of the electrical conductivity of Ti_3_C_2_T_*x*_ MXene films with various flake sizes. Data are presented as mean ± SD (*n* = 5). **f** Cyclic voltammetry curves of the various Ti_3_C_2_T_*x*_ MXene films at a scan rate of 20 mV s^–1^. **g** Stability of the Ti_3_C_2_T_*x*_ MXene with various flake sizes. The dotted lines represent fits based on the empirical equation in $A={A}_{unre}+{A}_{re}{e}^{-t/\tau }$. [Illustrations in **a** were created with BioRender.com]

As illustrated in Fig. 1a, epidermal electrodes are attached to the skin surface to monitor electrophysiological signals (including electromyography (EMG), electrocardiography (ECG), electrooculogram (EOG), and electroencephalogram (EEG)), which serve as crucial clinical indicators reflecting the physiological status of the human body [33]. The essence of electrophysiological signals lies in changes in the ionic concentration gradient across cell membranes. The acquisition of these signals involves the transduction of ionic currents into electronic signals via epidermal electrodes. Electron–ion transduction occurs at the electrode–skin interface. To understand the influence of electrode material properties and conformability on interfacial impedance, Fig. S1 illustrates electrical equivalent circuit models for three types of electrode–skin interfaces: wet gel electrodes, non-conformal dry electrodes, and conformal dry electrodes. As described in Note S1, wet gel electrodes have low interfacial impedance but suffer inherent limitations such as skin irritation and performance deterioration associated with gel dehydration [34]. In contrast, non-conformal dry electrodes exhibit air gaps between the electrode and skin, leading to a drastic increase in interfacial impedance [15]. Conformal dry electrodes closely conform to the complex topography of the skin surface and eliminate air gaps, thereby reducing interfacial impedance. The impedance of the conformal electrodes can be mathematically represented as:

5 $$Z(w) = \frac{{R_{d} }}{{1 + jwC_{d} R_{d} }} = \frac{{R_{d} }}{{1 + (wC_{d} R_{d} )^{2} }} - j\frac{{wC_{d} R_{d}^{2} }}{{1 + (wC_{d} R_{d} )^{2} }}$$

where *w* is the frequency [10, 35]. The impedance modulus can be expressed as:

6 $$|Z(w)| = \sqrt {\left( {\frac{{R_{d} }}{{1 + (wC_{d} R_{d} )^{2} }}} \right)^{2} + \left( {\frac{{wC_{d} R_{d}^{2} }}{{1 + (wC_{d} R_{d} )^{2} }}} \right)^{2} } = \sqrt {\frac{{R_{d}^{2} }}{{1 + (wC_{d} R_{d} )^{2} }}}$$

In the equation, all the component values are determined by the electrode materials and their geometry [7]. At a certain frequency, the high specific capacitance (*C*_*d*_) reduces the electrode impedance without increasing the electrode area for accurate electrophysiological monitoring [36, 37]. The effect arises because higher capacitance facilitates faster ion–electron transduction under alternating current conditions, thereby promoting more efficient transformation of ionic currents from electrophysiological signals into electronic currents. Therefore, the electron–ion interface requires excellent electrochemical performance to achieve low interfacial impedance, thereby ensuring the acquisition of high-fidelity electrophysiological signals.

### Fabrication and Characterization of HEC/MXene Epidermal Electrodes

To fabricate low-impedance epidermal electrodes, large Ti_3_C_2_T_*x*_ MXene nanosheets were synthesized to improve the conductivity. Two strategies were employed: i) increasing the size of Ti_3_AlC_2_ MAX precursors and ii) minimizing structural damage during the delamination process. The large Ti_3_AlC_2_ MAX sample was prepared preferentially using a particle sedimentation method (Fig. S2) [30]. SEM images revealed an increase in lateral size distribution from 9.2 μm (pristine MAX phase) to 21.2 μm (processed MAX phase) (Fig. S3). Multilayer Ti_3_C_2_T_*x*_ was obtained by selectively etching the Al layers from large Ti_3_AlC_2_ MAX precursors (Fig. S4). Vortex oscillation was used to mildly exfoliate large monolayer Ti_3_C_2_T_*x*_ MXene nanosheets by applying shear force to the surface of multilayer Ti_3_C_2_T_*x*_ (Fig. 1b) [38]. Comprehensive characterization confirmed the successful synthesis of large, monolayer Ti_3_C_2_T_*x*_ MXene nanosheets. Their delaminated two-dimensional morphology is clearly visible in the SEM image (Fig. 1c). TEM and elemental mapping (Fig. S5) further confirm the structural integrity and chemical uniformity of the flakes. The crystalline quality was demonstrated by the hexagonal symmetry observed in both high-resolution transmission electron microscopy (HRTEM) and selected area electron diffraction (SAED) pattern (Fig. S6). Crucially, AFM height profiling (Fig. S7) reveals a thickness of approximately 1.8 nm, consistent with the theoretical thickness of a single-layer Ti_3_C_2_T_*x*_ MXene [39]. The concentration of the large Ti_3_C_2_T_*x*_ MXene dispersion was approximately 12.9 mg mL^−1^, with a total yield of approximately 58%. The size distribution of the flakes was determined by statistical analysis of measurements from over 200 flakes across multiple SEM images (Fig. S8). The lateral size referred to the length (*x*, the longest distance) of the MXene flake. Quantitative size statistics (*x* and *y*) for the large Ti_3_C_2_T_*x*_ MXene are presented in Table S1. The mean lateral size (*x*) was 6.5 µm (Fig. 1d), and the mean width (*y*) was 3.2 µm (Fig. S9a), yielding an aspect ratio of approximately 2, consistent with previous literature reports [40].

For comparison, small and medium Ti_3_C_2_T_*x*_ MXene nanosheets were synthesized using different MAX phase and exfoliation methodologies (Note S2**)**. The small Ti_3_C_2_T_*x*_ MXene, prepared by ultrasonication, has a lateral size too small for reliable statistical analysis of its size distribution from SEM images. DLS results indicate a mean lateral size of approximately 0.43 µm (Fig. S9b). The medium Ti_3_C_2_T_*x*_ MXene exhibits a mean lateral size (*x*) of 4.6 µm and a mean width (*y*) of 2.6 µm (Fig. S9c, d). This comparative analysis demonstrates the effectiveness of the combined particle sedimentation and shear-force delamination method in producing larger MXene nanosheets. As shown in the AFM images in Fig. S10a, c, the thicknesses of the small and medium MXene nanosheets are 1.6 and 1.8 nm, respectively, which are comparable to that of the large MXene. Meanwhile, SEM images (Fig. S10b, d) reveal that the small MXene nanosheets prepared via ultrasonication exhibit surface holes, whereas the medium ones display a more intact surface. This indicates that shear-force delamination can effectively minimize structural damage to the MXene. The purity of the different MXene was determined by the absence of the 2*θ* peak of the MAX phase at ≈ 39° in the XRD patterns (Fig. S11a). The chemical structures of different MXene were characterized using ATR-FTIR spectroscopy, Raman spectroscopy, and XPS [41]. For MXene of different sizes, their spectral features are nearly identical, although the intensities of the characteristic peaks vary. This variation may arise from size-dependent effects, but requires further work to investigate. As shown in Fig. S11b, all three types of MXene exhibit characteristic peaks at 3473, 1640, and 622 cm^−1^, corresponding to the stretching vibrations of the -OH group, the C = O bonds, and the Ti–O bonds, respectively. In the Raman spectra (Fig. S11c), the intensity bands at 203 and 617 cm^−1^ are attributed to the in-plane vibrations of carbon atoms, while the bands in the spectral regions at 272, 420, and 510 cm^−1^ are assigned to the characteristic modes of Ti_3_C_2_(OH)_2_ MXene structures. It is worth noting that the intensity band at 150 cm^−1^ originates from laser-induced disruption of the MXene lattice [42]. The presence of surface terminal groups (-O, -OH, -F) can be further confirmed by XPS spectra (Fig. S12). As observed from the high-resolution Ti 2*p* spectrum, the peaks can be deconvoluted into four components corresponding to Ti, Ti^2+^, Ti^3+^, and TiO_2_ at binding energies of 454.7, 455.3, 456.2, and 459.2 eV, respectively [43]. Detailed fitting results for all peaks are provided in Table S2.

The correlation between the lateral size of MXene nanosheets and their electrical and electrochemical performance was systematically investigated using the freestanding films (≈ 8 μm thick) fabricated by vacuum-assisted filtration [44]. Figure S13 shows the surface morphologies of Ti_3_C_2_T_*x*_ MXene films with various flake sizes. The conductivity of Ti_3_C_2_T_*x*_ MXene films increases with increasing flake size, reaching a value 3.6 times higher in large MXene than in small MXene (Fig. 1e). This enhanced electrical conductivity can be attributed to the flatter surface and fewer basal-plane defects of large Ti_3_C_2_T_*x*_ MXene nanosheets (Fig. S10) [40]. Electrochemical measurements were conducted to probe the effect of lateral size on electrochemical performance. As shown in Fig. 1f, the CV curves of Ti_3_C_2_T_*x*_ MXene with various flake sizes all exhibit rectangular shapes, consistent with previous studies on Ti_3_C_2_T_*x*_ MXene [45]. The large Ti_3_C_2_T_*x*_ MXene film exhibits the highest capacitance (77.4 mF cm^−2^) compared to the medium (54.7 mF cm^−2^) and small (39.0 mF cm^−2^) counterparts. This discrepancy may originate from two factors. First is the difference in electrical conductivity. Large Ti_3_C_2_T_*x*_ MXene films possess higher electrical conductivity, which provides a more efficient electron transport network within the film. This facilitates the more complete and rapid utilization of active sites, a phenomenon that has been previously demonstrated in MXene materials [46]. Second is the difference in nanosheet defects. As shown in Fig. S10, nanosheets prepared via ultrasonication exhibit some defects (holes), whereas the large MXene nanosheets produced by the mild shear-force-assisted delamination method show fewer defects (Fig. 1c). This likely preserves more active sites in the large MXene nanosheets [47]. The increased capacitance contributes to reduced interfacial impedance in epidermal electrodes. The oxidative stability of various Ti_3_C_2_T_*x*_ MXene was investigated using UV–vis spectroscopy (Note S3 and Fig. S14a) [48]. As depicted in Fig. 1g, the absorbance intensity of MXene nanosheets decreased continuously over time. The experimental data were fitted with an empirical function [49], $A={A}_{unre}+{A}_{re}{e}^{-t/\tau }$, where ${A}_{unre}$ and ${A}_{re}$ correspond to the stable (unreactive) and unstable (reactive) fractions of MXene nanosheets, respectively, and τ represents the time constant (in days). The derived fitting parameters are summarized in Table S3. Large Ti_3_C_2_T_*x*_ MXene showed a slower decay rate and a larger time constant, indicating improved oxidative stability compared to small MXene. Furthermore, the time constant τ was plotted as a function of flake size *L* (μm) and fitted to the relationship: $\tau =5.4\times {10}^{0.021L}$ (Fig. S14b), highlighting the dependence of degradation behavior on flake size. Further XPS characterization was performed on different MXene nanosheets after stored for 10 days. The high-resolution Ti 2*p* spectra indicate that compared to the freshly prepared samples, a significant enhancement of the TiO_2_ peak at 459.2 eV was observed for the small MXene. A weaker enhancement was noted for the medium MXene, while the large MXene showed almost no increase (Fig. S15). These results indicate that large Ti_3_C_2_T_*x*_ MXene is more suitable for use as an epidermal electrode. Given its improved performance and stability, large MXene was selected for further investigation.

**Fig. 2:**
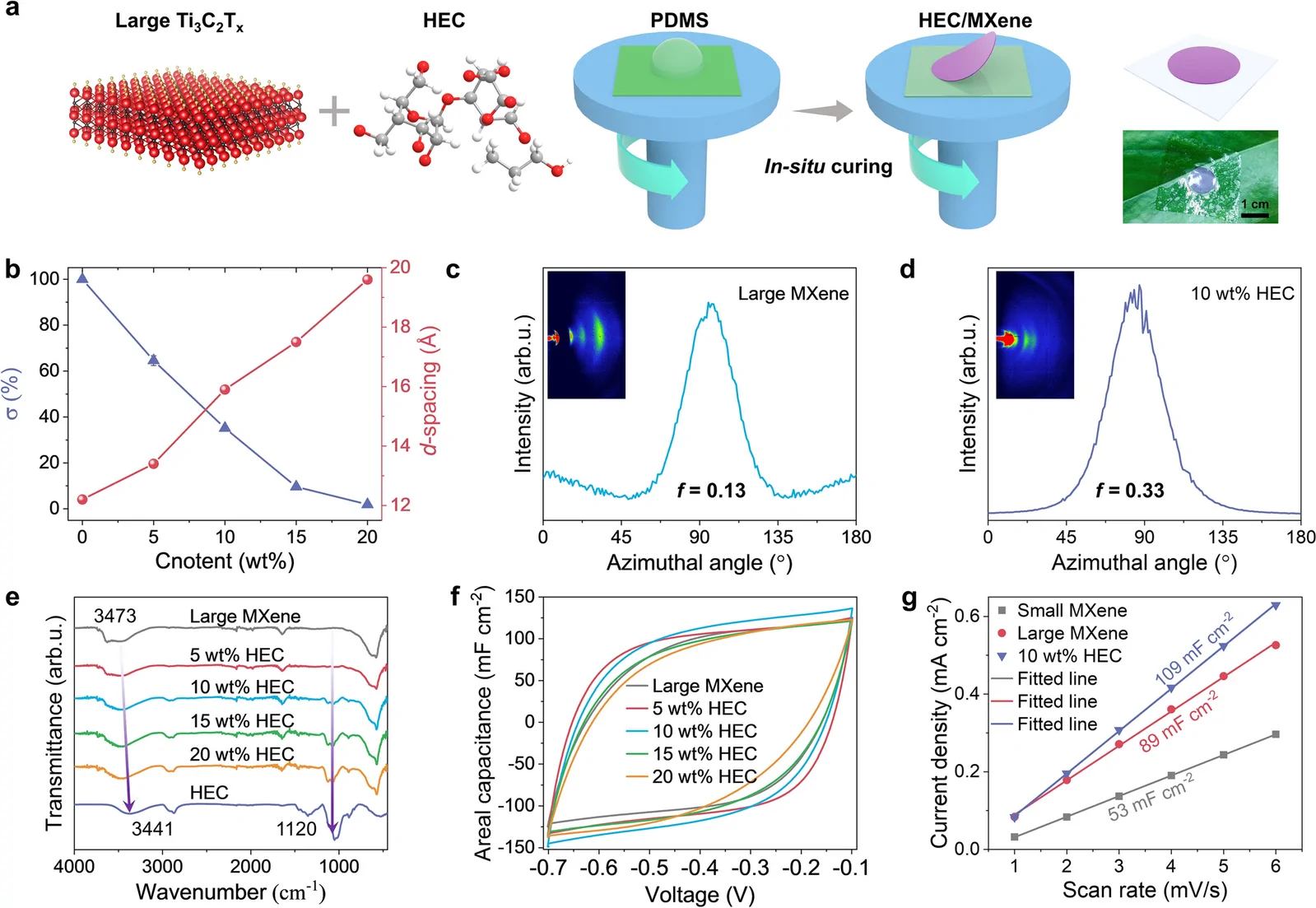
Preparation and performance optimization of HEC/MXene epidermal electrodes. **a** Schematic of the fabrication process for HEC/MXene epidermal electrodes. **b** Conductivities and *d*-spacing for large MXene and HEC/MXene films with various HEC loadings. Data are presented as mean ± SD (*n* = 5). **c, d** WAXS patterns and corresponding azimuthal plots of the (002) peak in the azimuth range of 0° and 180° for large MXene and the 10 wt% HEC/MXene films, demonstrating enhanced structural ordering upon HEC incorporation. **e** FTIR spectra of large MXene and HEC/MXene films with various HEC loadings, confirming the presence of HEC and its interaction with MXene. **f** Cyclic voltammetry curves measured in 1 × PBS at 20 mV s^−1^, showing the influence of HEC loading on electrochemical behavior. **g** Linear relationship between current and scan rate for small MXene, large MXene, and 10 wt% HEC/MXene films, indicating a dominant capacitive charge storage mechanism

As illustrated in Fig. 2a, HEC was introduced as a bridging agent to improve both the electrochemical performance and flexibility of large Ti_3_C_2_T_*x*_ MXene nanosheets for epidermal electrode applications [50, 51]. Film thickness increases with higher HEC loading at a fixed MXene amount (Table S4). The infrared image in Fig. S16 shows that the addition of HEC to the MXene dispersion did not induce any thermal effect, indicating that the compositing of HEC and MXene is a mild process. PDMS serves as a compliant substrate and is seamlessly integrated with HEC/MXene films through *in*-*situ* curing. The uncured PDMS layer, spin-coated onto a PET substrate, was covered with a patterned HEC/MXene layer. Following annealing for curing, the multilayer structure was peeled from the PET (Experimental Section). The PDMS layer (~ 0.1 mm thick) provides adhesion, enabling the epidermal electrode to conformally adhere to the skin surface [52]. The resulting HEC/MXene epidermal electrodes exhibit conformal adaptation to complex surface topographies, enabling reliable monitoring of high-fidelity electrophysiological signals.

The (002) diffraction peak in the XRD pattern evolved from 7.3° (large Ti_3_C_2_T_*x*_ MXene) to 4.5° with 20 wt% HEC incorporation (Fig. S17), corresponding to an interlayer *d*-spacing expansion from 12.1 to 19.6 Å (Fig. 2b). This enlarged interlayer spacing reduces steric effects, promoting ion diffusion kinetics and exposing more accessible active sites [50]. However, the electrical conductivity is reduced due to the intrinsic insulating nature of HEC. The expansion of interlayer *d*-spacing was further confirmed by high-angle annular darkfield (HAADF) scanning transmission electron microscopy (STEM) images (Fig. S18). The interlayer spacing of the large MXene increases from 1.26 to 1.64 nm upon incorporation of 10 wt% HEC. WAXS measurements were conducted to evaluate the effect of HEC molecules on the stacking of MXene nanosheets. As shown in Fig. 2c, d, Herman’s orientation factor (*f*) was employed to quantify the alignment of nanosheets relative to the film plane (Experimental Section). Compared to large MXene films (*f* = 0.13), 10 wt% HEC/MXene films exhibited a higher *f* = 0.33, indicating the formation of an orderly stacked structure. TEM images of the cross-sections of the films revealed that the large MXene nanosheets exhibit a disorderly stacking structure, whereas the 10 wt% HEC/MXene nanosheets display a more ordered arrangement (Fig. S19a, b). This well-aligned structure in 10 wt% HEC/MXene films is advantageous for enhancing both mechanical flexibility and electrochemical performance [52]. The interactions between large MXene nanosheets and HEC molecules were characterized by FTIR spectra (Fig. 2e). The -OH peak exhibits a redshift from 3473 to 3441 cm^−1^, with a concomitant decrease in intensity. As shown in Fig. S20, the XPS spectra of the HEC/MXene films show no new peaks compared with the large MXene film. The Ti 2*p* fitting results show that the peaks for Ti, Ti^2+^, and Ti^3+^ gradually shift to higher binding energies, from 454.69, 455.32, 456.15, and 459.21 eV for the large MXene to 454.89, 455.54, 456.38, and 459.32 eV at a 20 wt% HEC loading, respectively. Detailed fitting results are provided in Table S5. These results demonstrate the formation of weak hydrogen bonding between Ti_3_C_2_T_*x*_ MXene nanosheets and HEC molecules. As shown in Fig. S19c, the improved ordering of large MXene nanosheets may be attributed to hydrogen bonding interactions with HEC. The MXene nanosheets, which resemble rigid bricks, are prone to mutual obstruction during spontaneous stacking. In contrast, the soft HEC can act as a lubricant, guiding the MXene nanosheets into a more ordered arrangement through hydrogen bonding. The HEC/MXene films exhibit improved flexibility and mechanical stability. As shown in Fig. S21a, b, the HEC/MXene film demonstrates a smaller bending radius of 155 µm when bent to 180°, compared to the large MXene film (radius of 243 µm) [30]. After 500 cycles of repeated bending to 180°, the HEC/MXene film remained intact with negligible degradation in electrochemical performance (Fig. S21c, d). Notably, due to the inorganic nature of MXene, the electrodes are not stretchable.

The electrochemical performance is governed by synergistic electron and ion transport processes. Although HEC incorporation reduces electrical conductivity, it simultaneously promotes interlayer expansion and improves nanosheet alignment. These structural modifications facilitate ion transport while exposing more active sites. Figure 2f displays the CV profiles of large MXene and HEC/MXene films with various HEC loadings. The capacitance of the films increases upon HEC incorporation, reaching a maximum 91.8 mF cm^−2^ at 10 wt% HEC loading (Fig. S22). The enhanced capacitance arises from accelerated ion transport and increased accessibility of active sites in HEC/MXene films, enabled by their expanded interlayer spacing and orderly nanosheet stacking. The capacitance reduction at higher HEC loadings results from the insulating nature of HEC. Although the introduction of HEC reduced the conductivity to 1081 S cm^−1^, this value remains higher than that of conventional conductive polymers [53, 54]. Consequently, the 10 wt% HEC/MXene was selected for further application in epidermal electrodes. To investigate the capacitance mechanism, the double-layer capacitance at the electrode–electrolyte interface was calculated from the linear relationship between current and scan rate in the narrow non-Faraday region of the CV (Fig. S23). The 10 wt% HEC/MXene film exhibits a double-layer capacitance of 109 mF cm^−2^, which is higher than that of its counterparts (Fig. 2g) [36]. The enhanced electrochemical performance and flexibility contribute to effectively reducing interfacial impedance in epidermal electrode applications [55]. The high capacitance of this electrode suggests its potential for electrical stimulation applications. To evaluate this capability, voltage transients on the electrodes were measured while delivering charge-balanced, biphasic current pulses of 1 mA, with a pulse duration of 500 μs for both the cathodic (*t*_c_) and anodic (*t*_a_) phases and an inter-pulse interval (*t*_ip_) of 250 μs. As shown in Fig. S24, the electrode demonstrates efficient charge-transfer capability, and no significant change was observed after 100 stimulation cycles, indicating good stability. Furthermore, as shown in Fig. S25, the CV curves of the HEC/MXene film were tested over a wide potential range (-0.7 to + 0.4 V) and consistently maintained rectangular shapes, indicating purely double-layer capacitive behavior without any redox reactions. This also ensures that no potentially harmful electrochemical side reactions, which could damage the electrode or the skin, occur during electrophysiological signal acquisition. To assess practical applicability, CV was also measured in artificial sweat, a more skin-relevant electrolyte (Fig. S26). Although the absolute current density was different within PBS, the performance ranking of all electrodes remained consistent, confirming the validity of the electrodes. The uniformity of the 10 wt% HEC/MXene electrode material across different synthesis batches was confirmed by analyzing the elemental ratio using XPS. As shown in Fig. S27, the Ti/O atomic ratio remains nearly identical across three batches. These results confirm that HEC/MXene can serve as an epidermal electrode.

### Performances of HEC/MXene Epidermal Electrodes

**Fig. 3:**
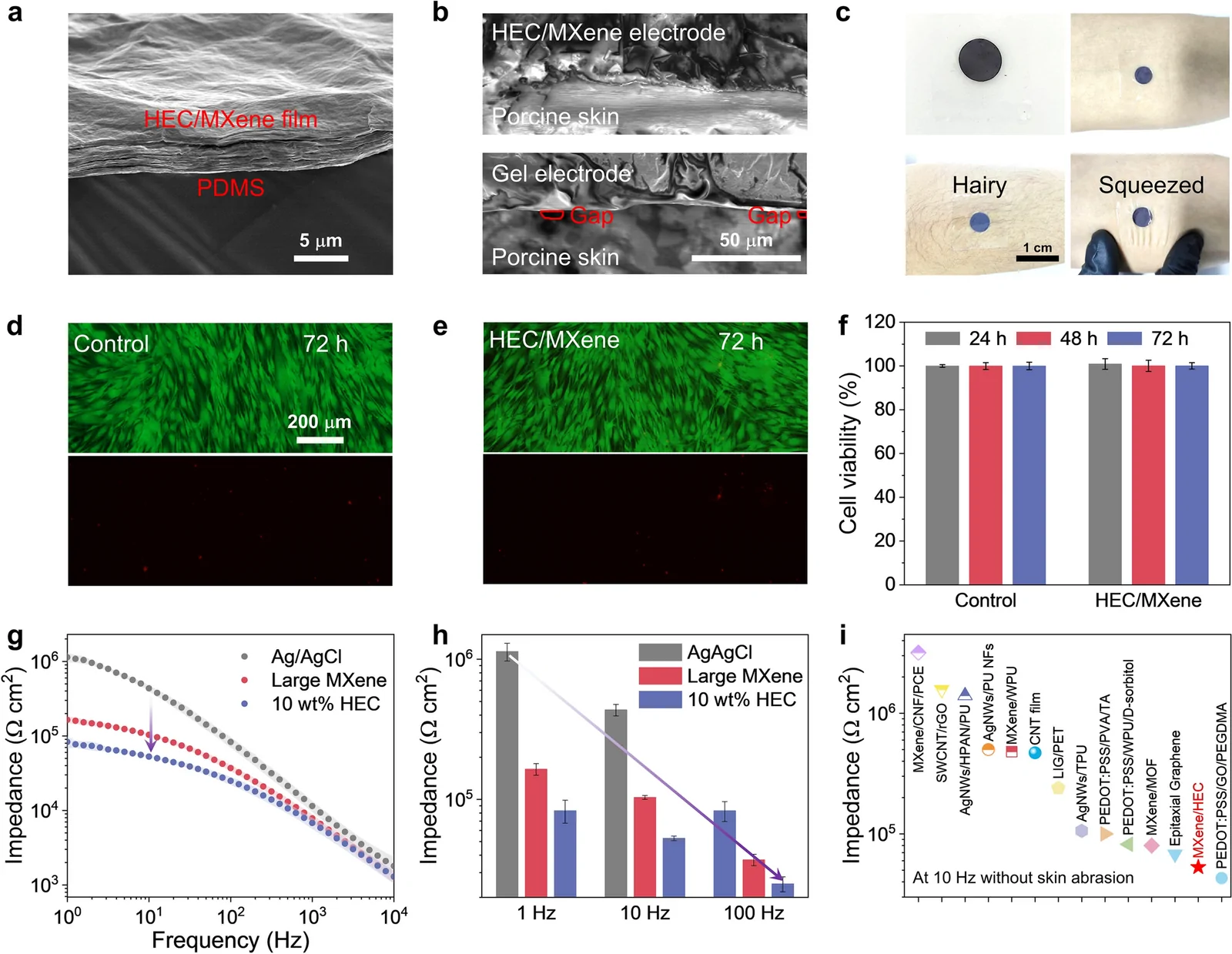
Comprehensive evaluation of HEC/MXene electrodes: conformability, biocompatibility, and interfacial impedance. **a** Cross-section SEM image showing the structure of the electrode. **b** SEM images of the interface between the porcine skin and different electrodes, demonstrating conformal contact. **c** Optical images showing conformability on different skins (smooth, hairy, squeezed). **d, e** Fluorescence microscopy images (green: viable cells, red: dead cells) of live/dead human skin fibroblast cells for control and HEC/MXene film groups after 72 h immersion. **f** Cell viability of control and HEC/MXene film groups after 24, 48, and 72 h. Data are presented as mean ± SD (*n* = 3). **g** EIS spectra and **h** the corresponding interfacial impedance for commercial Ag/AgCl gel, large MXene, and HEC/MXene electrodes on the skin. Data are presented as mean with shaded regions corresponding to SD (*n* = 5). Data recorded from *N* = 1 subject. **i** Performance comparison of HEC/MXene electrodes with previously reported epidermal electrodes at 10 Hz without skin abrasion

As shown in Fig. 3a, cross-sectional SEM images of the HEC/MXene electrode reveal a robust interfacial bond between the HEC/MXene film and the PDMS substrate, which enhances the conformability of epidermal electrodes. The robust interfacial bond between the HEC/MXene film and PDMS likely originates from hydrogen bonding crosslinking between the abundant surface terminal groups (-OH, -O) on the MXene surface and the hydroxyl groups present in the uncured PDMS. The adhesion forces of the epidermal electrodes on skin were evaluated by a standard 90° peel test (Fig. S28a). The adhesion force approached 0.2 N cm^−1^ on skin (Fig. S28b). Therefore, the HEC/MXene epidermal electrode conformally attaches to the porcine skin surface without any gaps. In contrast to the commercial gel Ag/AgCl electrode (the clinical gold standard for electrophysiology recording), which exhibits visible gaps at the skin interface (Fig. 3b), the HEC/MXene electrodes maintain conformal contact with diverse skin surfaces, including smooth, hairy, and deformed skin (Fig. 3c). The cytotoxicity of HEC/MXene epidermal electrodes was assessed using HSF cells to evaluate their biocompatibility for practical epidermal electrophysiological applications. Fluorescent live/dead staining revealed minimal dead cells (indicated by red fluorescence) in both the control group and the 10 wt% HEC/MXene film group (Figs. 3d, e, and S29). Moreover, cells cultured with the 10 wt% HEC/MXene film exhibited comparable viability to those in the control group at 24, 48, and 72 h (Fig. 3f), demonstrating excellent biocompatibility for wearable applications. It is worth noting that the degradation products of Ti_3_C_2_T_*x*_ are primarily titanium dioxide and amorphous carbon, which are generally considered inert and non-toxic. This alleviates environmental concerns; however, their long-term impacts still warrant continued investigation [42, 56].

The on-skin biocompatibility of the HEC/MXene epidermal electrodes was evaluated through comparative assessment with commercial Ag/AgCl gel electrodes. The HEC/MXene epidermal electrodes induced neither irritation nor inflammation after 24 h of wear (Fig. S30). Meanwhile, the infrared image in Fig. S31 shows that the temperature of the HEC/MXene epidermal electrode is consistent with that of the skin, and no heat accumulation was observed after continuous wear for 1 h, indicating that the electrode possesses good conformability and heat dissipation properties. The electrode–skin interfacial impedance was measured using electrochemical impedance spectroscopy (EIS) on the forearm, with electrodes placed on the skin surface (Fig. S32). To enable direct comparison, impedance values were normalized by the electrode area. The HEC/MXene epidermal electrodes exhibit lower interfacial impedance than both large and small MXene electrodes (Figs. 3g and S33). Meanwhile, across the frequency range of 1–1000 Hz, the interfacial impedance is consistently lower than that of commercial Ag/AgCl gel electrodes (Fig. 3h). At 10 Hz, their interfacial impedances are 53 ± 19 and 436 ± 21 kΩ cm^2^, respectively. Most electrophysiological signals fall within this frequency range (Table S6), enabling the HEC/MXene epidermal electrodes to accurately monitor various electrophysiological signals [7]. As summarized in Table S7, we compiled typical impedance values of Ag/AgCl gel electrodes reported in the literature at 10 Hz. The impedance generally ranged from 252 to 3150 kΩ cm^2^. The values for the commercial Ag/AgCl gel electrodes used in this study are consistent with those reported in the literature, with observed variations likely due to differences in gel composition across commercial brands. Although Ag/AgCl gel electrodes establish rapid ion transport pathways through the gel medium, they lack high interfacial capacitance. Moreover, the gel medium typically exhibits low conductivity, which limits its capability for efficient ion–electron transduction under alternating current conditions.

The impedance of HEC/MXene electrodes is lower than that of previously reported advanced epidermal electrodes (Fig. 3i and Table S8). Compared with highly conductive electrodes, which exhibit reduced electrode–skin interfacial impedance despite relatively low conductivity, this performance advantage arises because interfacial impedance depends not only on conductivity but also on the electrochemical performance of the electrode materials. The HEC/MXene electrodes exhibit excellent capacitance combined with high conductivity, resulting in low interfacial impedance. To further demonstrate the benefit of enhanced capacitive behavior, EIS was fitted using the above equivalent circuit model (Fig. 1a). As shown in Fig. S34, 10 wt% HEC/MXene exhibits a higher double-layer (*C*_*d*_) than large MXene, which is consistent with the previous CV test results.

### Recording and Analysis of EMG Signals

**Fig. 4:**
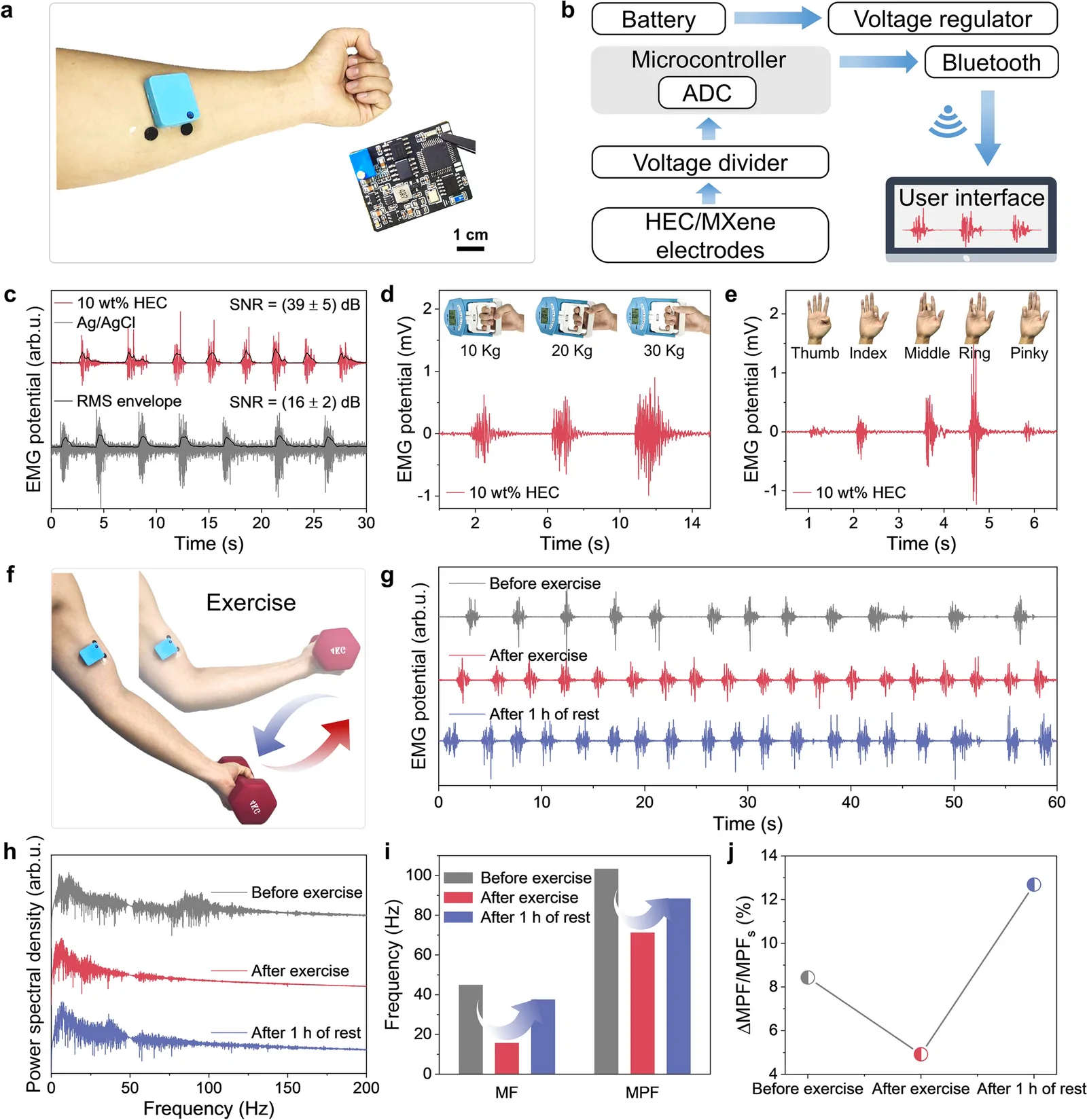
Portable EMG monitoring system and signal analysis for muscle activity and fatigue assessment. **a** Photographic image of the PCB and miniature recording system. **b** System-level block diagram of the transmission from electrodes to the user interface. **c** EMG signals acquired by commercial Ag/AgCl gel electrodes and 10 wt% HEC/MXene epidermal electrodes. Data are presented as mean ± SD (*n* = 8 trials). The root-mean-square (RMS) envelope of the EMG signal is overlaid in black. **d, e** EMG signals under various gripping strengths and isolated finger flexion. **f** Photograph of the exercise. **g** EMG signals, **h** power spectral density (PSD), and **i** median frequency (MF) and mean power frequency (MPF) extracted before exercise, immediately after exercise, and after 1 h of rest. **j** Corresponding rate of change in MPF, evaluating the level of muscle fatigue. Data recorded from *N* = 1 subject

EMG signals have become increasingly important for wearable applications and HMIs. To facilitate portable signal monitoring, a miniaturized recording system was integrated using a reusable printed circuit board (PCB) (Fig. 4a). The data acquisition circuit and PCB diagram are shown in Fig. S35. The microcontroller unit (MCU) utilized is the STM32F103C8T6, which integrates an analog-to-digital converter (ADC). The system-level block diagram in Fig. 4b illustrates the electrophysiological signal flow. Signals from the epidermal electrodes are acquired using a voltage divider and the ADC, then wirelessly transmitted to a user device via Bluetooth for various applications. As shown in Fig. S36a, two HEC/MXene epidermal electrodes were placed on the flexor muscles. The HEC/MXene epidermal electrodes exhibited a 2.4-fold enhancement in SNR (39 ± 5 dB) compared to commercial Ag/AgCl gel electrodes (16 ± 2 dB) (Fig. 4c). As shown in Table S9, for different subjects, although the SNR shows some variation due to individual differences, it consistently remains higher than that of Ag/AgCl gel electrodes. Additionally, the HEC/MXene epidermal electrodes were applied for long-term EMG monitoring. As shown in Fig. S37, the electrodes maintained low skin interface impedance and high SNR after 48 h.

The accurate detection of EMG signals enables the use of HEC/MXene epidermal for muscle strength and gesture recognition [57]. When a dynamometer was gripped with various forces (10, 20, and 30 kg), the generated EMG signals were monitored with the HEC/MXene epidermal electrodes (Fig. 4d). Furthermore, these electrodes can recognize gestures by measuring EMG signals generated during the flexion of different fingers (Fig. 4e). The root-mean-square (RMS) values of the EMG signal amplitudes are consistent with the gripping forces and gestures (Fig. S38). These features are distinguishable without the need for classification algorithms; however, achieving finer gesture recognition and practical application may require the integration of machine learning algorithms for classification. The assessment of muscle fatigue status is valuable for exercise guidance. Muscle activity monitoring was performed by placing two HEC/MXene epidermal electrodes on the biceps brachii muscles (Fig. 4f). Figure 4g presents EMG signals obtained before exercise, immediately after physical exertion (isolated curls and push-ups), and after a 1-h recovery period. The average power spectra of the EMG signals exhibited increased amplitudes, indicating muscle fatigue. Meanwhile, decreases in both median frequency (MF) and MPF were also observed, further confirming muscle fatigue [58]. Figure 4j displays the relative variations in MPF across distinct muscle conditions [59]. The most pronounced relative change in MPF was observed following exercise and a 1-h recovery period, indicating propensity for muscular fatigue during the immediate post-exercise period. This result provides valuable insights for optimizing training protocols.

### Accurate Monitoring of ECG, EOG, and EEG Signals

**Fig. 5:**
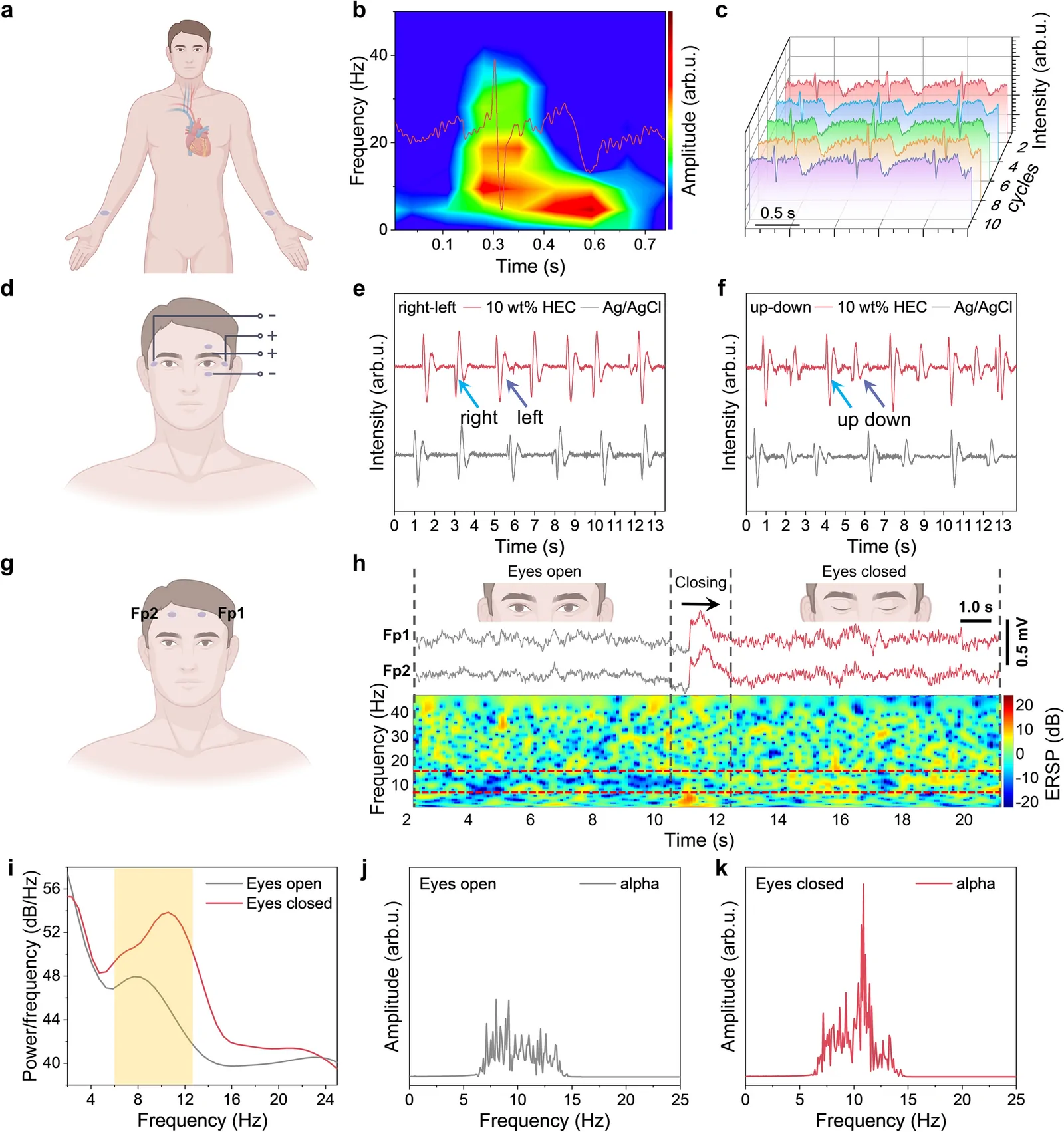
Diverse physiological signal monitoring using the HEC/MXene epidermal electrodes. **a** Schematic of the ECG configuration. **b** Time–frequency spectrogram of ECG signal monitored by HEC/MXene epidermal electrodes. **c** Multiple cycles monitoring of ECG signal with HEC/MXene epidermal electrodes. **d** Schematic of the electrode placement for EOG monitoring. **e, f** EOG signals correspond to eye movements of right-left and up-down. **g** Schematic of the frontal electrode placement. **h** Original EEG signals and their Fast Fourier Transform (FFT) spectrogram during eyes-open and eyes-closed states. **i** Corresponding power spectral density (PSD), highlighting the increase in alpha band (8–13 Hz) power upon eye closure. **j, k** Extracted alpha wave components during eyes-open and eyes-closed states. Data recorded from *N* = 1 subject. [Illustrations in **a**, **d**, **g**, and **h** were created with BioRender.com]

ECG is a crucial electrophysiological signal for the diagnosis and treatment of cardiovascular diseases. With low interfacial impedance and conformability, HEC/MXene epidermal electrodes can serve as wearable electrodes for ECG monitoring. Two HEC/MXene epidermal electrodes were affixed to the forearm to record ECG signals (Figs. 5a and S36b). Figure S39a shows the ECG signals detected by both types of electrodes, in which the P, QRS, and T waveforms are distinguished. The HEC/MXene epidermal electrode exhibits a peak‑to‑peak QRS voltage (0.6 mV) comparable to that of the Ag/AgCl electrode (Fig. S39b). The spectrogram of the ECG rhythm was acquired using the fast Fourier transform (FFT), revealing distinguishable PQRST complexes within distinct frequency ranges, a feature critical for diagnosing cardiovascular diseases (Fig. 5b) [53]. It is worth noting that the extraction of these features is sufficient for daily health monitoring. For complex disease-related characteristics, advanced data analysis may be required in consultation with medical professionals. As shown in Fig. S40, the HEC/MXene epidermal electrodes can be used for monitoring ECG signals under various temperature and relative humidity (RH) conditions encountered in daily life. The HEC/MXene epidermal electrodes can be used for multi-cycle ECG monitoring. As shown in Fig. 5c, ECG signals maintained high quality after continuous monitoring for 10 cycles. The electrode‑skin interface impedance exhibited no significant degradation, the QRS voltage of the ECG signals remained stable, and the baseline RMS noise showed only minor fluctuations, demonstrating the durability of the electrode through multiple reuse cycles (Fig. S41).

Eye movements are a promising modality for HMI [60]. High-precision EOG is an effective method for detecting eye movements [61]. EOG signals were recorded by attaching HEC/MXene epidermal electrodes around the orbital region, as illustrated in Figs. 5d and S36c. The left and right electrodes were connected to a single channel to capture horizontal eye movement signals (Fig. 5e). The vertical electrode pair was positioned to record vertical ocular movements and blink-related potentials (Figs. 5f and S42). The EOG signals acquired by HEC/MXene electrodes showed higher peak-to-peak amplitudes than those recorded with commercial Ag/AgCl gel electrodes across all eye movements, indicating their potential application in eye movement tracking. Recording high-fidelity EEG signals is more difficult due to their inherently weak signal strength with amplitudes in the microvolt range and interference originating from the scalp. Given low electrode–skin interfacial impedance, HEC/MXene epidermal electrodes enabled accurate EEG signal monitoring. Two HEC/MXene epidermal electrodes were positioned on the frontal region (Fp1 and Fp2) according to the 10–20 international EEG system (Figs. 5g and S36d). EEG signals were recorded during both eyes-open and eyes-closed states, and a prominent 10 Hz alpha rhythm was observed during the eyes-closed state (Fig. 5h). This rhythm was characterized by a higher power spectral density (PSD) than that observed in the eyes-open state (Fig. 5i). The amplitude of the extracted alpha wave in the eyes-closed state was higher than that in the eyes-open state (Fig. 5j, k). This alpha wave signal is one of the most widely studied and reliable behavioral EEG signals [62]. Furthermore, variations in the frequency distribution range of high-frequency EEG beta waves under different states were also observed (Fig. S43). EEG experiments demonstrated that HEC/MXene epidermal electrodes can accurately monitor EEG signals.

## Conclusions

In conclusion, the electrochemically enhanced low-impedance HEC/MXene dry epidermal electrodes were fabricated for accurate electrophysiological monitoring. The incorporation of large MXene nanosheets with HEC molecules, which form hydrogen bond bridges, resulted in expanded interlayer spacing and an orderly stacking structure within the HEC/MXene epidermal electrodes. This configuration endows the electrodes with both excellent electrochemical performance and high conductivity. The robust interfacial bond established between the HEC/MXene film and the PDMS substrate through *in*-*situ* curing enables the epidermal electrode to achieve conformal contact with skin. Consequently, the electrodes achieve reduced interfacial impedance, enabling higher signal quality than commercial Ag/AgCl gel electrodes and other epidermal electrodes in the literature. Moreover, a miniature recording system was integrated with HEC/MXene epidermal electrodes, which can transmit the signals wirelessly to the user interface. They can be used to record high-fidelity electrophysiological signals such as EMG, ECG, EOG, and EEG, with subsequent applications in gesture recognition and healthcare. This work provides promising material and regulation strategies for developing low-impedance dry epidermal electrodes.

## Supplementary Information

Below is the link to the electronic supplementary material.

Supplementary file1 (DOCX 9810 KB)
