# Supplementary material for: Electrochemically Enhanced Low-Impedance Ti3C2Tx MXene Epidermal Electrodes for Accurate Electrophysiological Monitoring
Source: Nanomicro Lett. 2026 Mar 20;18:297. doi: 10.1007/s40820-026-02132-9 (PMC13004779; doi:10.1007/s40820-026-02132-9)
Supplement: Supplementary file 1 — Supplementary file1 (DOCX 9810 KB) [file 40820_2026_2132_MOESM1_ESM.docx]

Supporting Information for

**Electrochemically Enhanced Low-Impedance Ti_3_C_2_T*_x_* MXene Epidermal Electrodes for Accurate Electrophysiological Monitoring**

Liubing Fan^1,2^, Fangfang Gao^1,2^, Liangxu Xu^1,2^, Xiaochen Xun^1,2^, Shuchang Zhao^1,2^, Bing Yang^1,2^, Han Bi^1,2^, Xuan Zhao^1,2^*, Qingliang Liao^1,2^*, Yue Zhang^1,2^*

^1^ Academy for Advanced Interdisciplinary Science and Technology, Beijing Advanced Innovation Center for Materials Genome Engineering, University of Science and Technology Beijing, Beijing 100083, P. R. China

^2^ Key Laboratory of Advanced Materials and Devices for Post-Moore Chips, Ministry of Education, Beijing Key Laboratory for Advanced Energy Materials and Technologies, School of Materials Science and Engineering, University of Science and Technology Beijing, Beijing 100083, P. R. China

*Corresponding authors. E-mail: [xuanzhao@ustb.edu.cn](mailto:xuanzhao@ustb.edu.cn) (Xuan Zhao); [liao@ustb.edu.cn](mailto:liao@ustb.edu.cn) (Qingliang Liao); [yuezhang@ustb.edu.cn](mailto:yuezhang@ustb.edu.cn) (Yue Zhang)

Note S1 Electrical equivalent circuit models for different electrode-skin interfaces

The dermal and subcutaneous layers can be represented by a resistor (*R_t_*), with a capacitor (*C_e_*) and a resistor (*R_e_*) connected in parallel, both of which simulate the epidermis [S1]. The potential (*E_se_*) arises from different ion concentrations across the stratum corneum. At the interface of the electrode and electrolyte, a half-cell potential (*E_hc_*) between the electrode and tissue is presented. The double-layer structure of the electrode-skin interface is modeled by a parallel circuit comprising a capacitor *C_d_* and a resistor *R_d_*, where *R_d_* represents the charge transfer resistance across the interfacial layers. For wet gel electrodes employed in commercial products, which utilize electrolyte gels, the equivalent circuit model incorporates the gel resistance (*R_g_*). Owing to skin irritation concerns associated with electrolyte gels, dry electrodes have inherent advantages. For non-conformal dry electrodes, there are air gaps between the electrode and skin, which can be modeled by another capacitor (*C_gap_*) and resistor (*R_gap_*) placed in parallel. This additional parallel *RC* circuit of air gaps will drastically increase the electrode-skin interfacial impedance [S2].

**Note S2 Preparation of diverse Ti_3_C_2_T*_x_* MXene nanosheets**

The traditional sonication exfoliation method, when applied to pristine MAX precursors, yielded MXene nanosheets with a mean lateral size of approximately 0.43 μm (denoted as small MXene). In contrast, vortex oscillation exfoliation of the pristine MAX resulted in MXene nanosheets, demonstrating a substantially larger mean lateral dimension of ~4.6 μm (denoted as medium MXene, Fig. S7). Consequently, the shear force method effectively facilitates the delamination of Ti_3_C_2_T*_x_* MXene nanosheets. Notably, when applying vortex oscillation exfoliation to processed MAX precursors, the resultant MXene nanosheets achieved exceptional lateral dimensions averaging ~6.5 μm (denoted as large MXene, **Fig. 1**d).

Note S3 Oxidation stability of diverse Ti_3_C_2_T*_x_* MXene nanosheets

The UV-vis spectra analysis of Ti_3_C_2_T*_x_* MXene nanosheets with various flake sizes (Fig. S11a) reveals distinct features at 325 nm, showing size-dependent characteristics consistent with electronic structure variations between edge sites and basal plane [S2, S3]. A comparatively gradual decline of normalized UV-vis spectra indicates the slower degradation rate of large Ti_3_C_2_T*_x_* MXene (**Fig. 1**g), where the points were fitted by an empirical function [S4], $A=A_{unre}+A_{re}e^{-t/\tau}$, $\tau$ is the time constant (days) and the fitting parameters are provided in Table S2. The $\tau$ can be plotted as a function with the mean flake size *L* (μm) and fitted using the following formula: $\tau=5.4\times{10}^{0.021L}$ (Fig. S14b), which demonstrates the size effect and proves that degradation initiates at the edges [S3].

**Supplementary Figures and Tables**


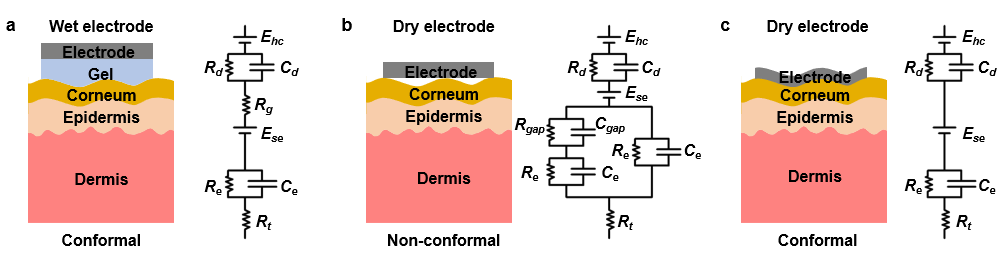


Fig. S1 Schematic and equivalent circuit models of three typical electrode-skin interfaces. (**a**) Wet gel electrodes. (**b**) Non-conformal dry electrodes. (**c**) Conformal dry electrodes


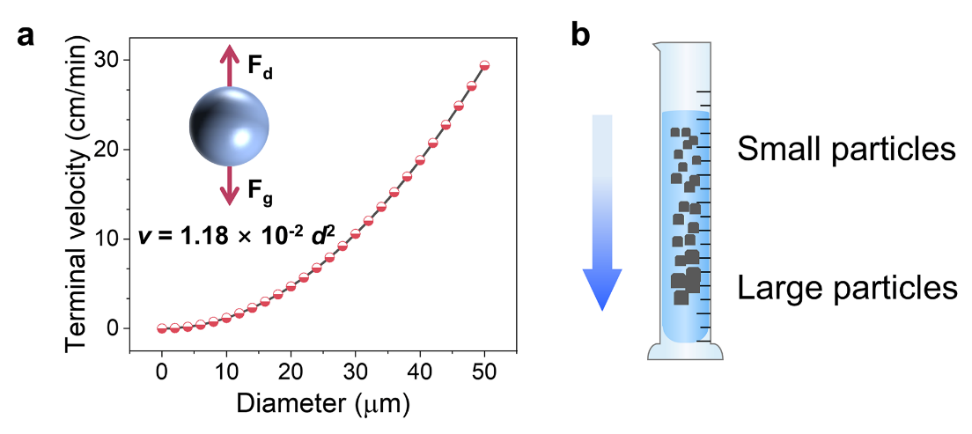


Fig. S2 Schematic of the sedimentation method for large MAX particles. (**a**) Mathematical model of particle sedimentation. (**b**) Schematic illustration of different particles in solution


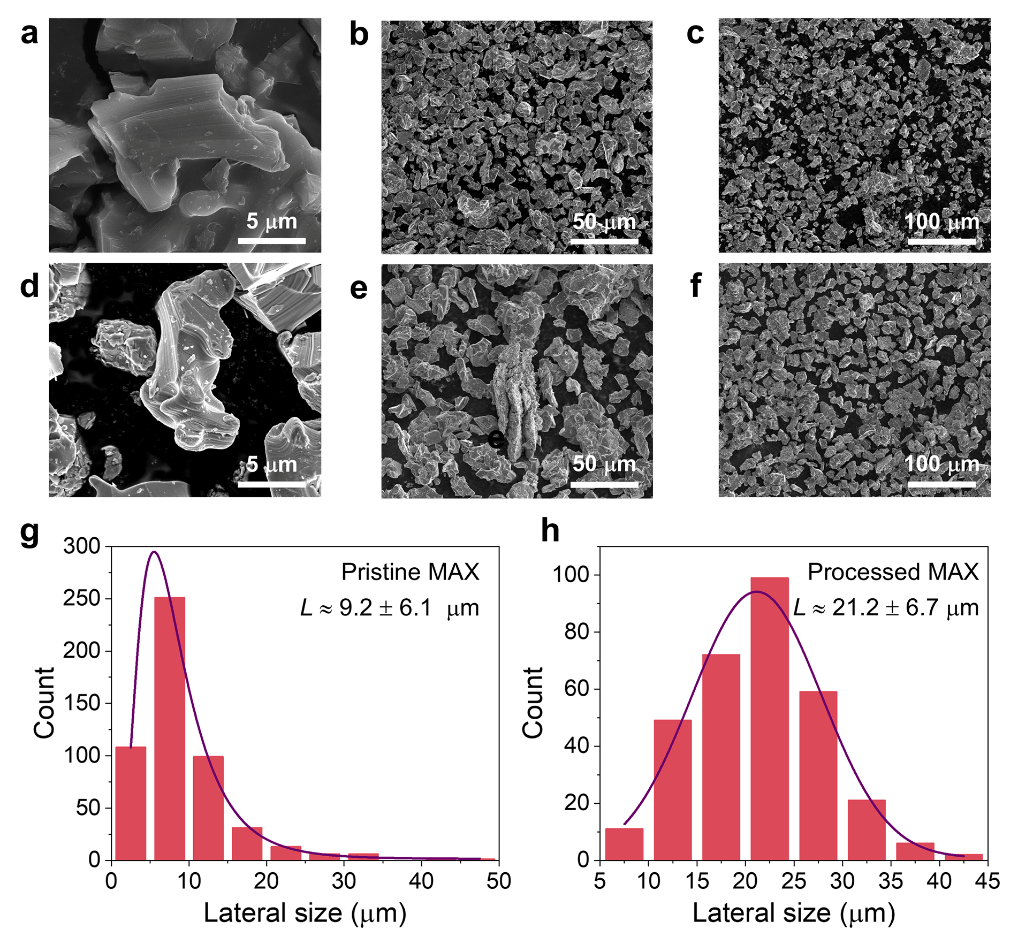


Fig. S3 Particle lateral size statistics of pristine and processed Ti_3_AlC_2_ MAX phase. (**a**), (**b**), and (**c**) SEM images of pristine Ti_3_AlC_2_ MAX phase. (**d**), (**e**), and (**f**) SEM images of processed Ti_3_AlC_2_ MAX phase after sedimentation. Lateral size distribution of (**g**) pristine Ti_3_AlC_2_ and (**h**) processed Ti_3_AlC_2_ MAX phase


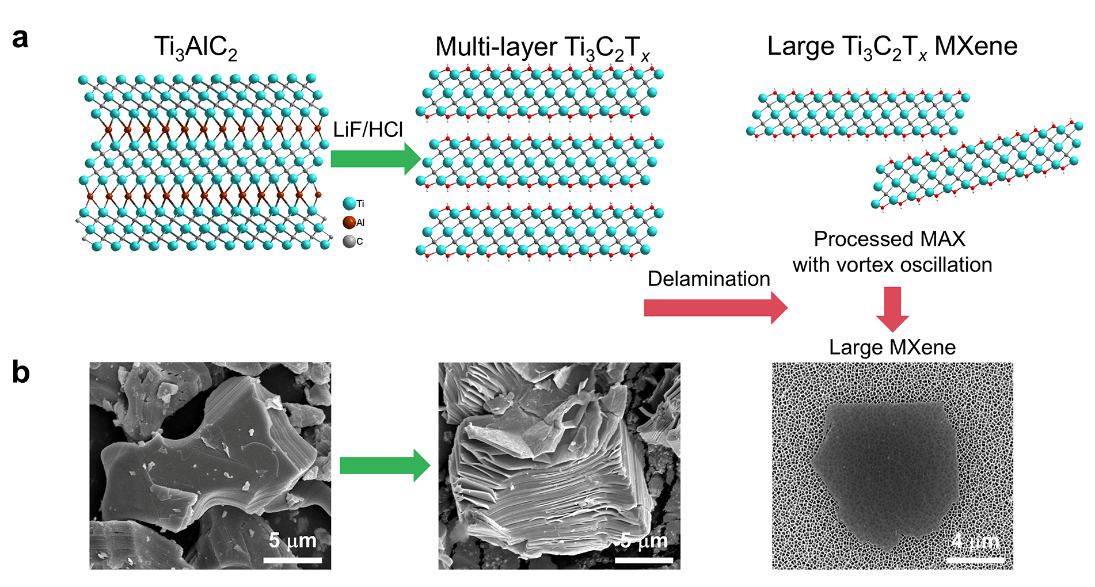


Fig. S4 Synthesis and structural evolution process of large MXene nanosheets. (**a**) Schematic illustration of the process flow from MAX phase to delaminated nanosheets. (**b**) SEM images of MAX, multi-layer MXene, and few-layer MXene nanosheet


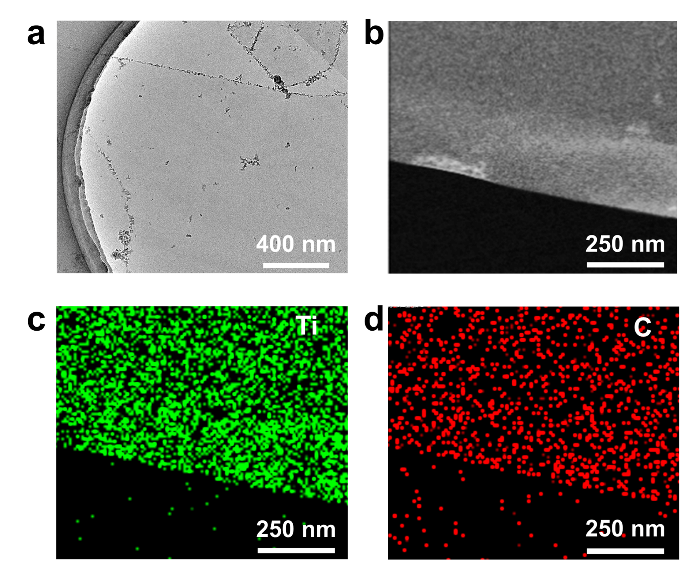


Fig. S5 TEM analysis and elemental mapping of large Ti_3_C_2_T*_x_* MXene nanosheets. (**a**), (**b**) TEM images of large Ti_3_C_2_T*_x_* MXene nanosheets. Elemental mapping of (**c**) Ti and (**d**) C


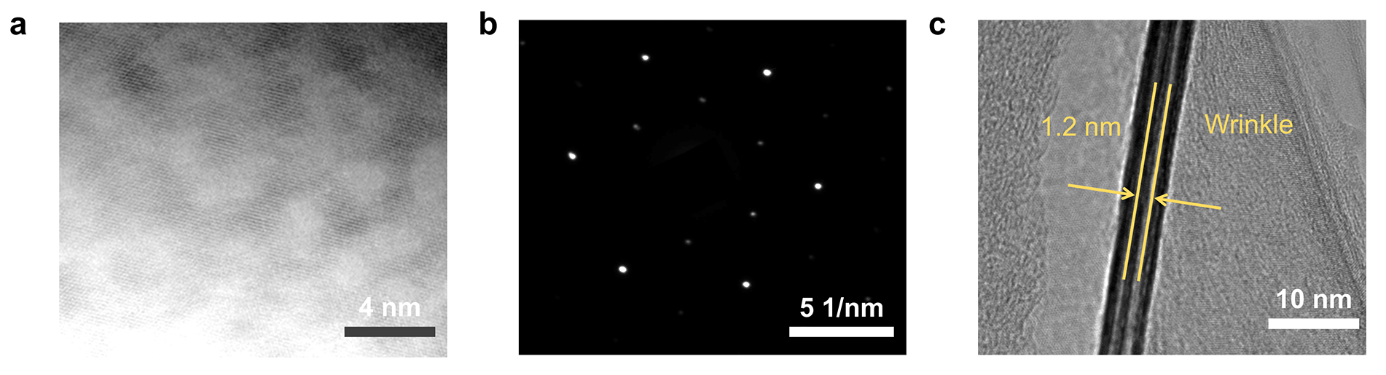


Fig. S6 TEM images of large MXene nanosheets. (**a**) High-resolution transmission electron microscopy (HRTEM) image and (**b**) corresponding selected area electron diffraction (SAED) pattern. (**c**) TEM image showing the MXene layers with an interlayer spacing of 1.2 nm


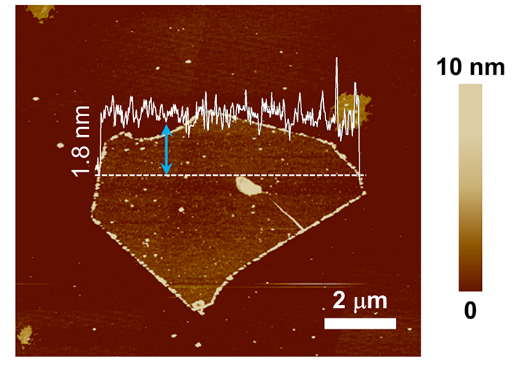


**Fig. S7** AFM image of the large Ti_3_C_2_T*_x_* MXene nanosheet


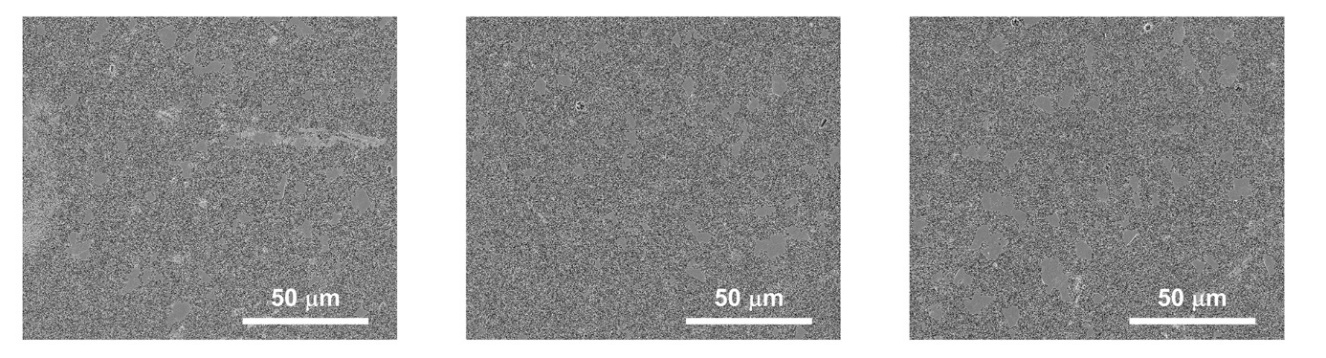


**Fig. S8** Multiple SEM images of large MXene nanosheets for statistical analysis of lateral size distributions


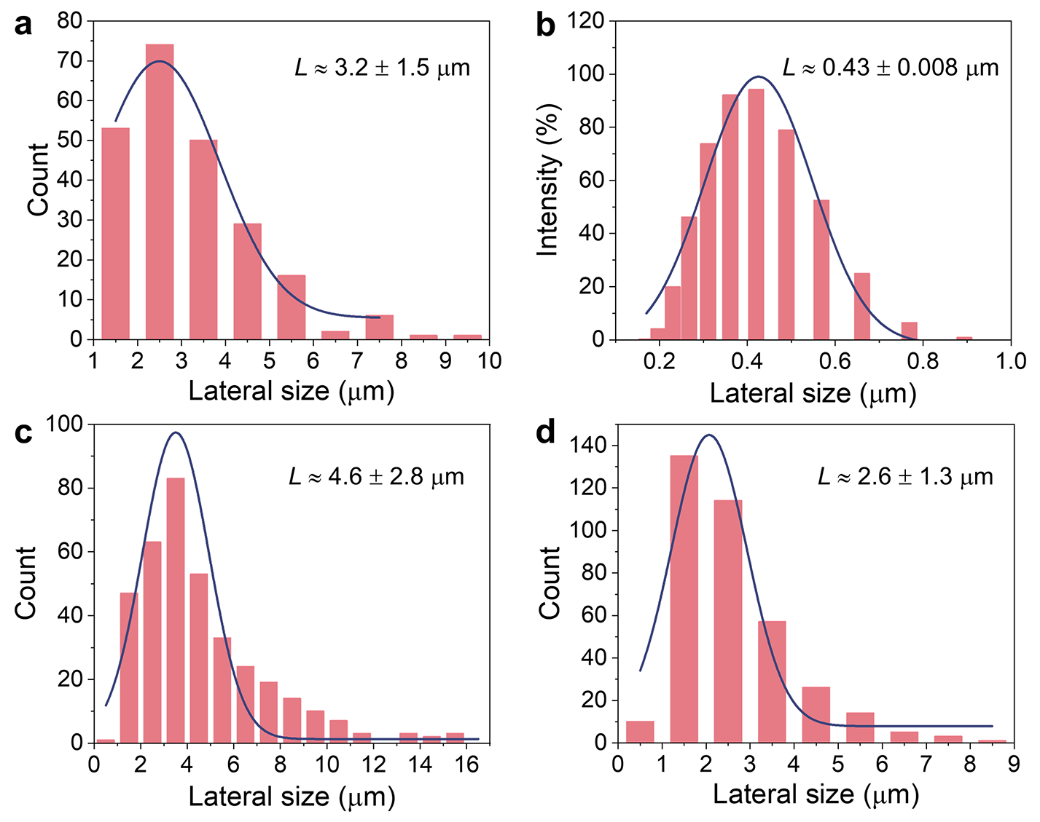


Fig. S9 Statistical distribution of the size of various Ti_3_C_2_T*_x_* MXene. (**a**) The width (*y*) flake size of large Ti_3_C_2_T*_x_* MXene. (**b**) Dynamic light scattering (DLS) data of small Ti_3_C_2_T*_x_* MXene. (**c**), (**d**) The length (*x*) and width (*y*) of medium Ti_3_C_2_T*_x_* MXene


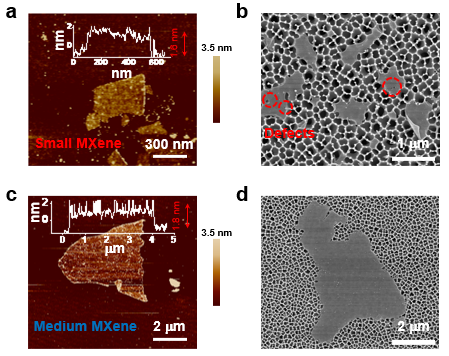


Fig. S10 Morphological characterization of Ti_3_C_2_T*_x_* MXene nanosheets with different flake sizes. (**a**) AFM image and (**b**) SEM image of small Ti_3_C_2_T*_x_* MXene nanosheets. (**c**) AFM image and (**d**) SEM image of medium Ti_3_C_2_T*_x_* MXene nanosheets.

Based on the AFM images, the thicknesses of small MXene and medium MXene nanosheets are 1.6 and 1.8 nm, respectively. These values exceed the theoretical monolayer thickness of Ti_3_C_2_, which is due to the existence of H_2_O molecules and surface oxidation [S5, S6].


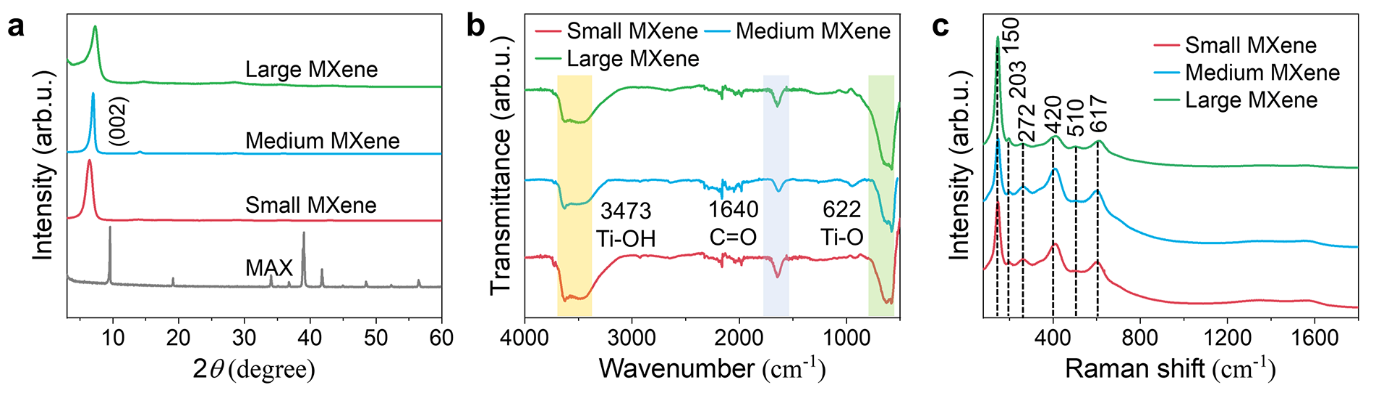


Fig. S11 Chemical composition and structure characterization of Ti_3_C_2_T*_x_* MXene films with various flake sizes. (a) XRD patterns of Ti_3_AlC_2_ MAX phase and Ti_3_C_2_T*_x_* MXene films with various flake sizes. (**b**) FTIR and (**c**) Raman spectra of Ti_3_C_2_T*_x_* MXene films with various flake sizes

The purity of synthesized Ti_3_C_2_T*_x_* MXene nanosheets with various flake sizes was demonstrated by the disappearance of the Ti_3_AlC_2_ MAX phase peak at 2*θ* = 39° in the XRD pattern. The chemical structure and composition of Ti_3_C_2_T*_x_* MXene with various flake sizes were confirmed using attenuated total reflection FTIR spectroscopy, Raman spectroscopy, and XPS, which indicated a consistent skeletal Ti-C structure and the presence of surface groups (-O, -OH, and -F) [S7].


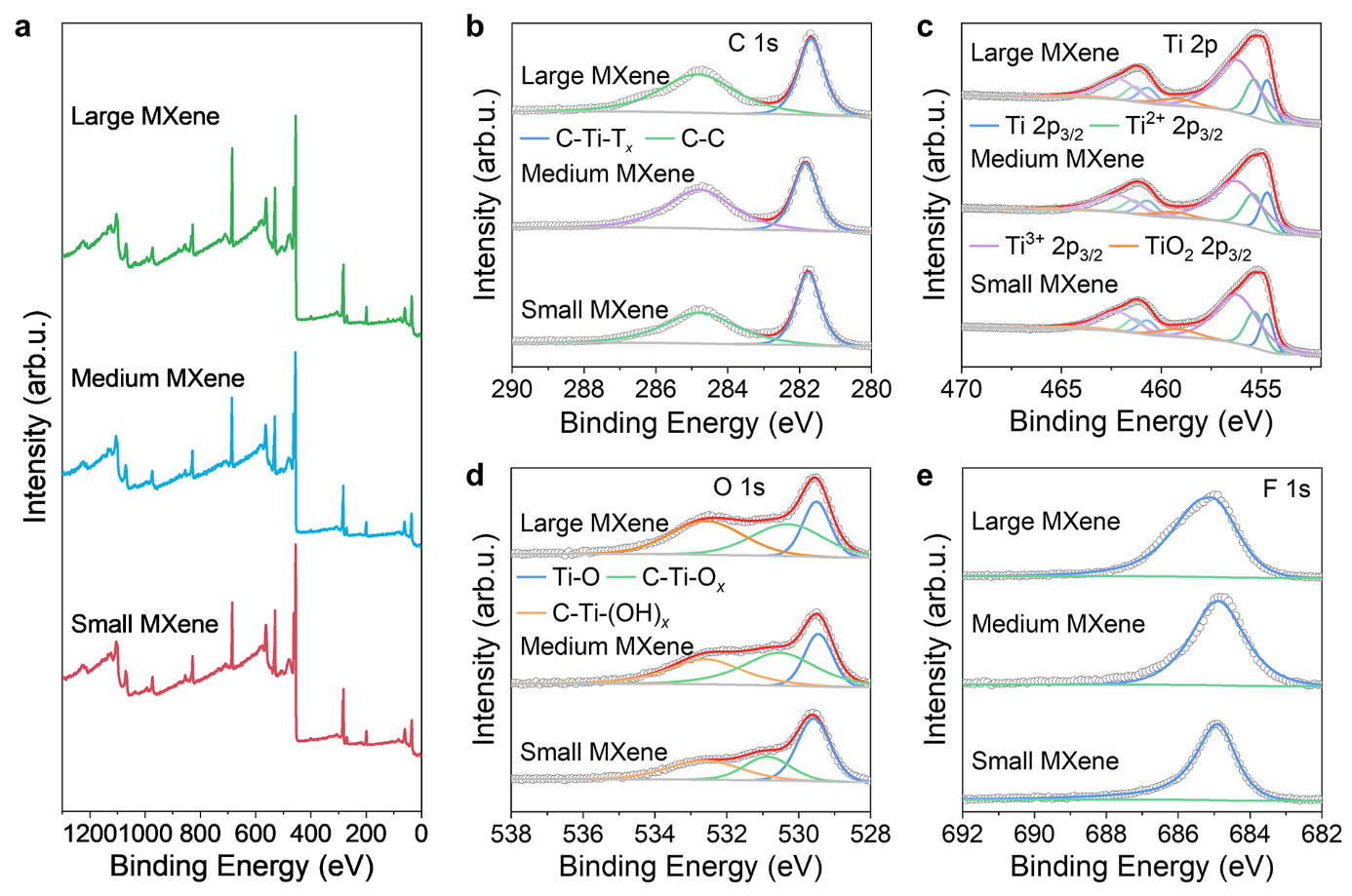


Fig. S12 XPS spectra characterization of Ti_3_C_2_T*_x_* MXene films with various flake sizes. (**a**) XPS survey, (**b**) C 1s, (**c**) Ti 2p, (**d**) O 1s, and (**e**) F 1s spectra


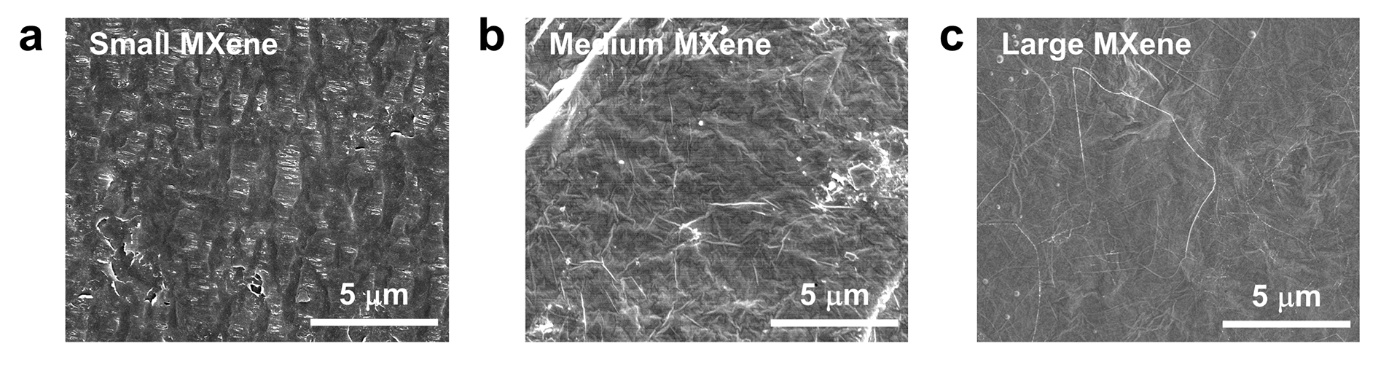


Fig. S13 Morphological characterization of Ti_3_C_2_T*_x_* MXene films with various flake sizes. SEM images of surface morphology for (**a**) small, (**b**) medium, and (**c**) large Ti_3_C_2_T*_x_* MXene films

The Ti_3_C_2_T*_x_* MXene films with various flake sizes demonstrate significant morphological differences. As the flake size increases, the surfaces of Ti_3_C_2_T*_x_* MXene films become progressively flatter.


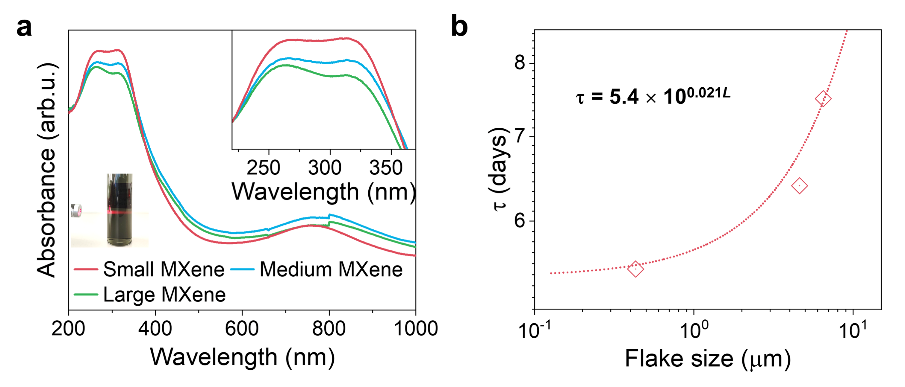


Fig. S14 UV-vis spectra characterization of Ti_3_C_2_T*_x_* MXene nanosheets with various flake sizes. (**a**) Normalized UV-vis spectra of Ti_3_C_2_T*_x_* MXene dispersions with various flake sizes (inset is the photograph showing the Tyndall effect of the large Ti_3_C_2_T*_x_* MXene dispersion). (**b**) The function of $\boldsymbol{\tau}$ and flake size, the dotted line shows the fitted results


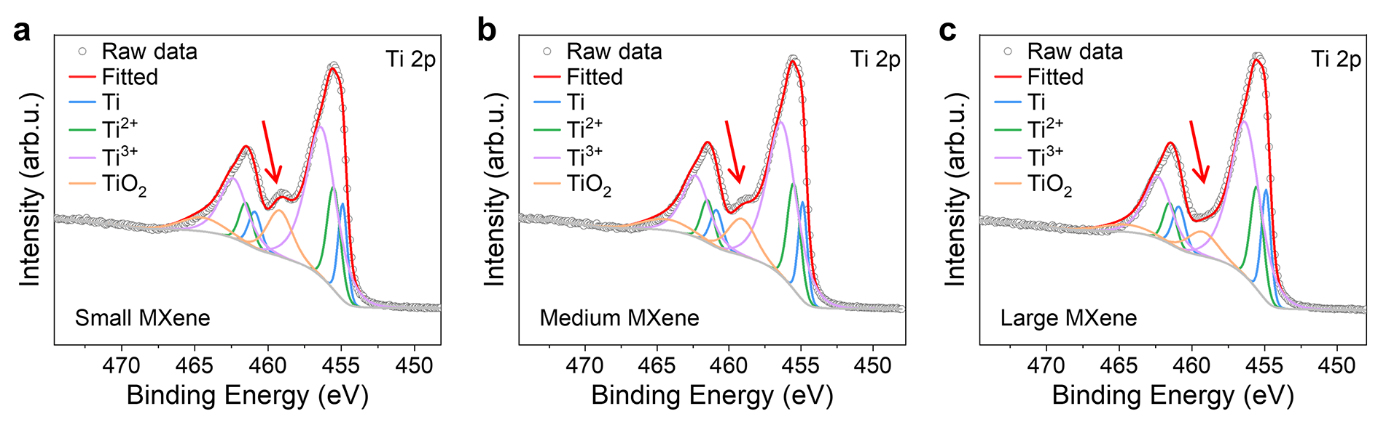


Fig. S15 Ti 2p XPS spectra of different MXene nanosheets after storage for 10 days. (**a**) Small MXene. (**b**) Medium MXene. (**c**) Large MXene


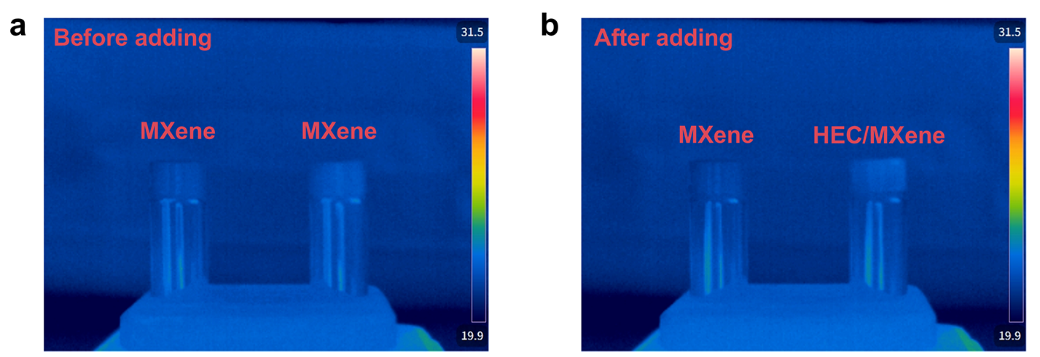


Fig. S16 The infrared images of MXene and HEC/MXene. (**a**) Before adding HEC. (**b**) After adding HEC


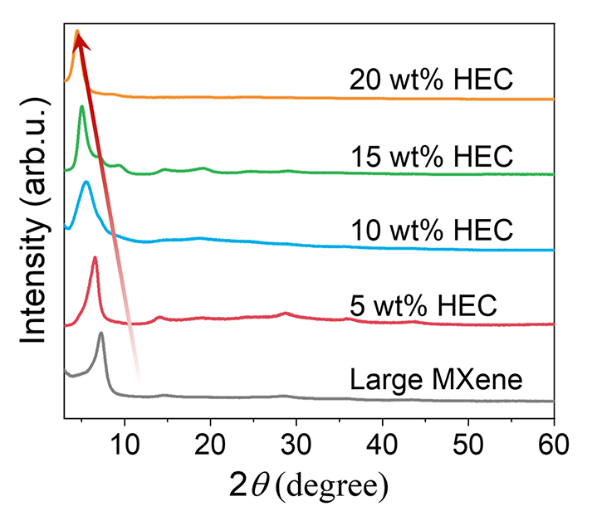


Fig. S17 XRD patterns of large MXene and HEC/MXene films with various HEC loadings


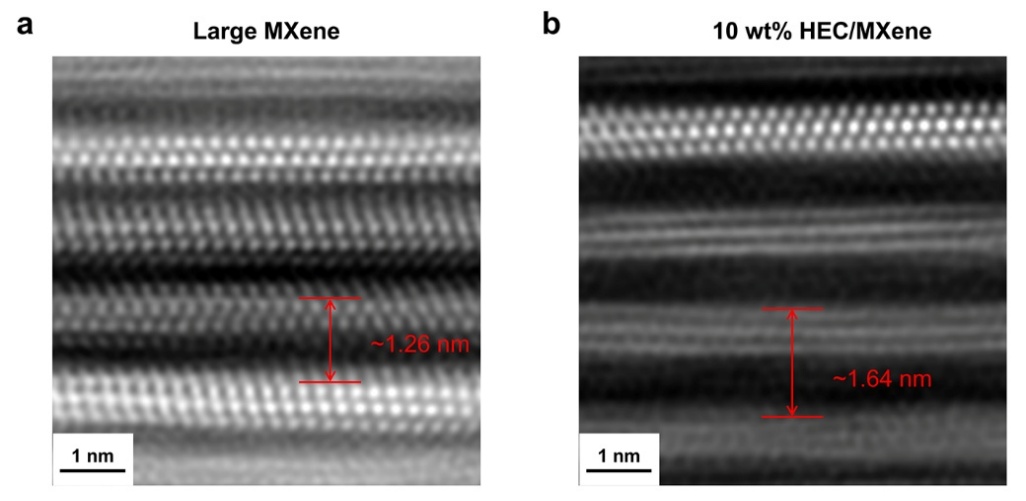


Fig. S18 High-angle annular darkfield (HAADF) scanning transmission electron microscopy (STEM) images of (**a**) large MXene with an interlayer spacing of 1.26 nm and (**b**) 10 wt% HEC/MXene with an interlayer spacing of 1.64 nm


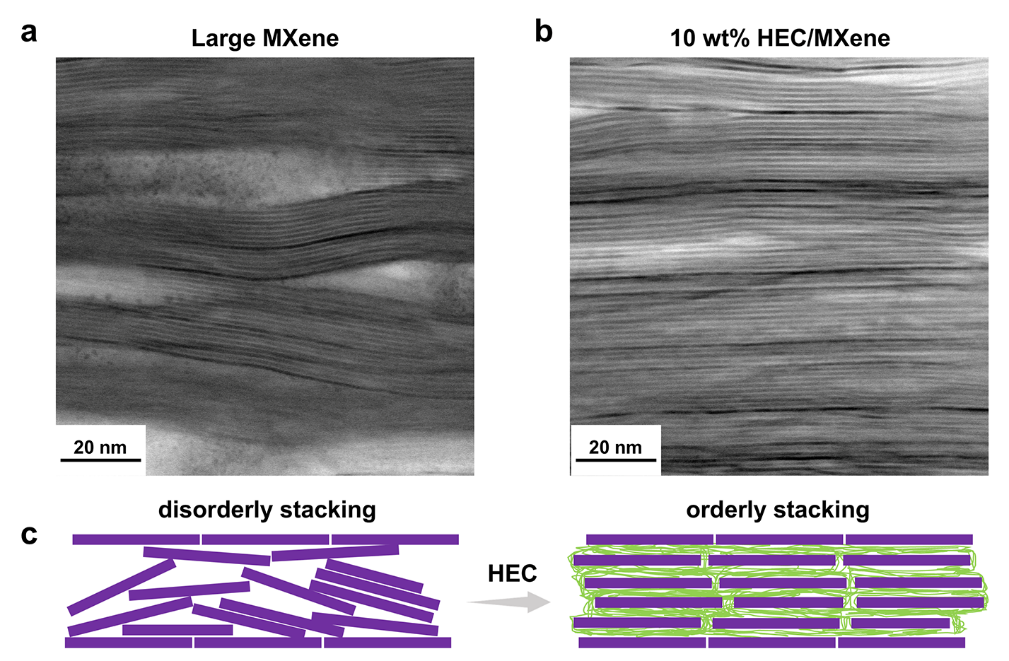


Fig. S19 The MXene nanosheets stacking morphology of the cross-sections of films. (**a**) TEM image of the large MXene film with a disorderly stacking structure. (**b**) TEM image of 10 wt% HEC/MXene film with orderly stacking structure. (**c**) Schematic illustration of the stacking structure of large MXene and 10 wt% HEC/MXene film


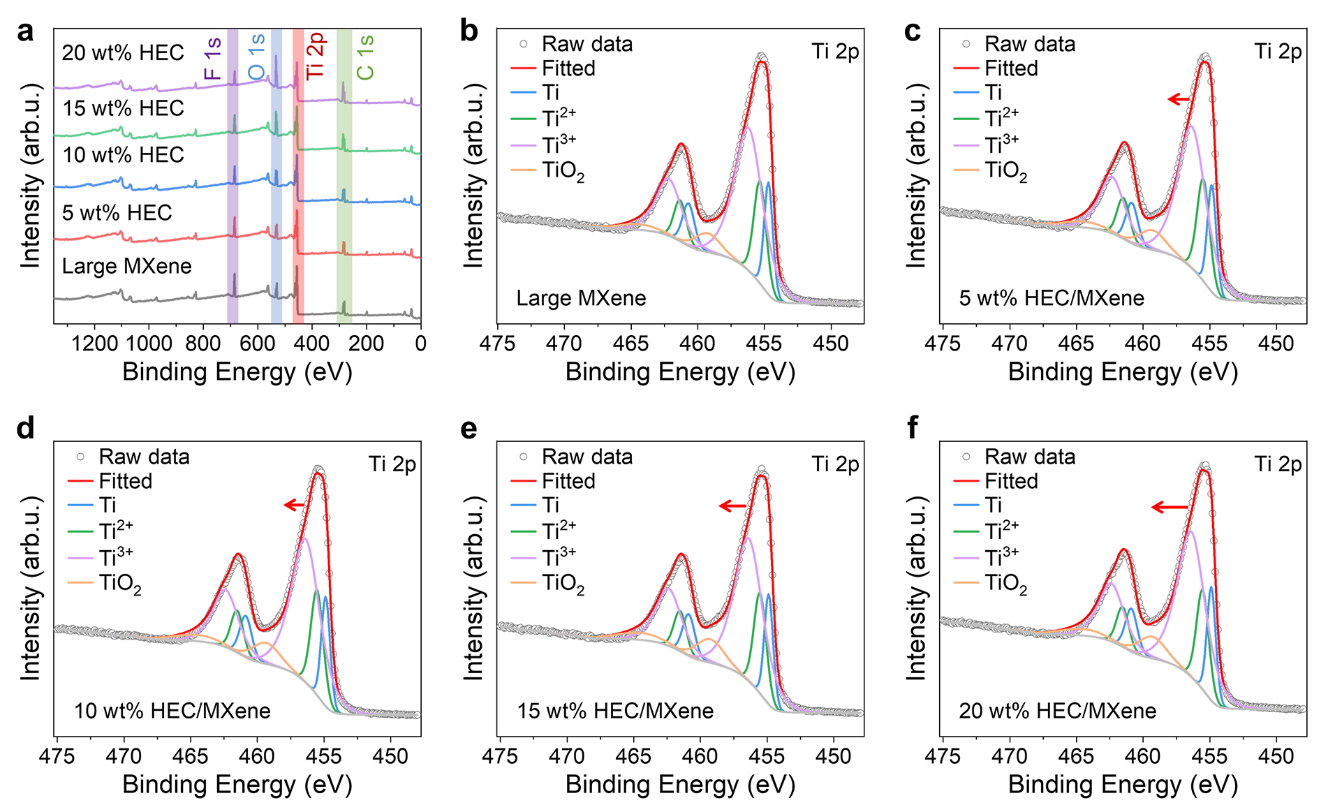


Fig. S20 XPS survey and Ti 2p spectra of large MXene and HEC/MXene films with various HEC loadings. (**a**) XPS survey. Ti 2p spectra of (**b**) Large MXene, (**c**) 5 wt% HEC/MXene, (**d**) 10 wt% HEC/MXene, (**e**) 15 wt% HEC/MXene, and (**f**) 20 wt% HEC/MXene


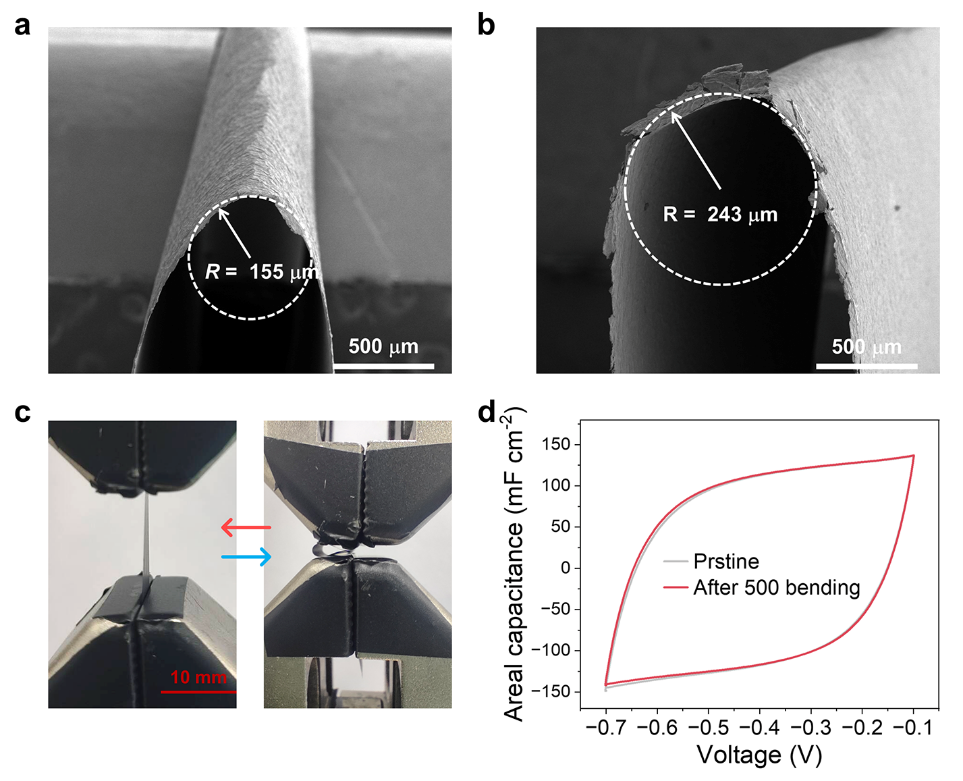


Fig. S21 Flexibility and mechanical durability of large MXene and HEC/MXene films. (**a**) SEM image illustrating an HEC/MXene film folded 180° with a bending radius of ~155 μm. (**b**) SEM image illustrating a large MXene film folded 180° with a bending radius of ~243 μm. (**c**) Photos showing an HEC/MXene film (15 mm × 12 mm) during a bending cycle. (**d**) Electrochemical performance of the HEC/MXene film before and after 500 bending cycles


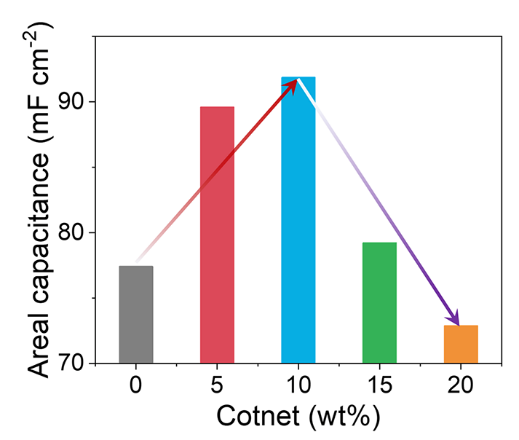


Fig. S22 Capacitances of large MXene and HEC/MXene films with various HEC loadings


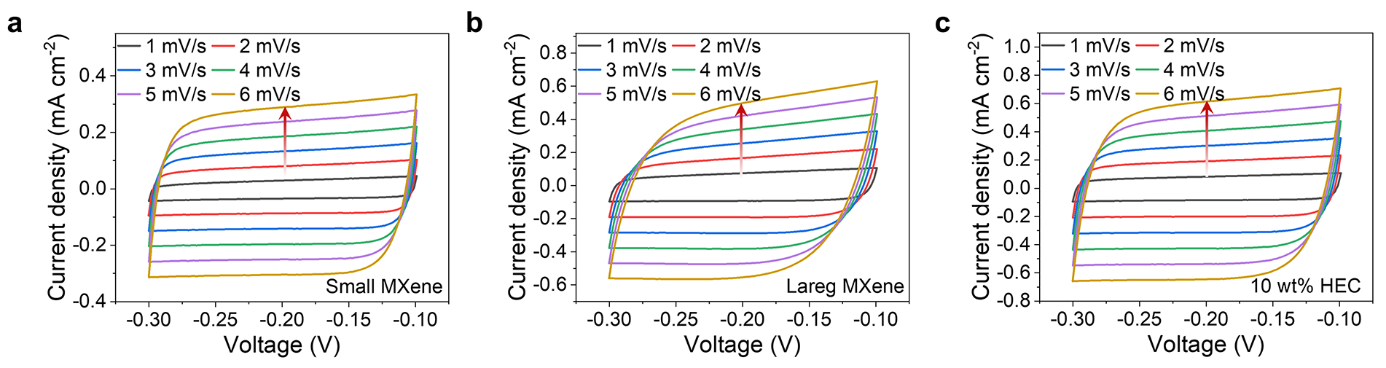


Fig. S23 Electrochemical characterization of small MXene, large MXene, and 10 wt% HEC/MXene films. cyclic voltammetry curves measured in 1× PBS for (**a**) small MXene, (**b**) large MXene, and (**c**) 10 wt% HEC/MXene films in the region of -0.3 ~ -0.1 V with scan rates of 1-6 mV/s


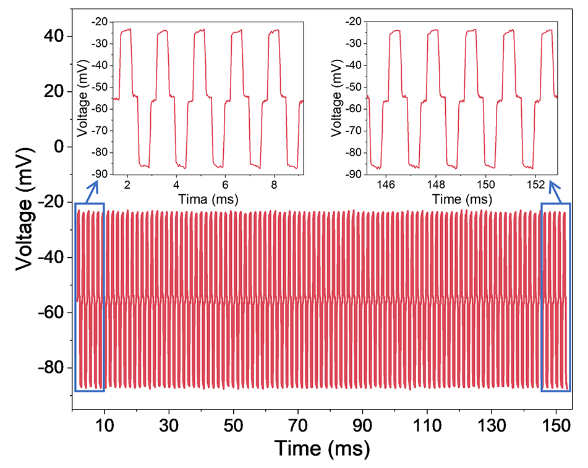


**Fig. S24** The 100 stimulation cycles of charge-balanced pulses of 1 mA for 10 wt% HEC/MXene film in 1× PBS


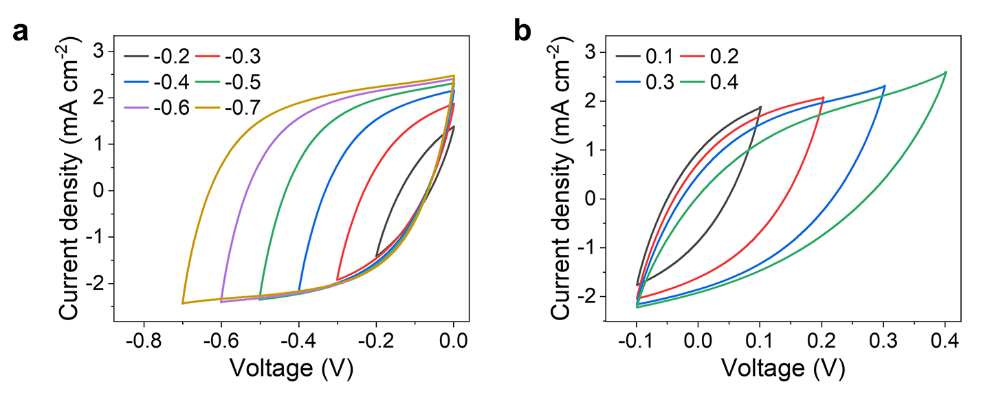


Fig. S25 Cyclic voltammetry (CV) curves of HEC/MXene film with scan rates of 20 mV/s in 1× PBS. (**a**) CVs exploring the negative voltage limit. (**b**) CVs exploring the positive voltage limit


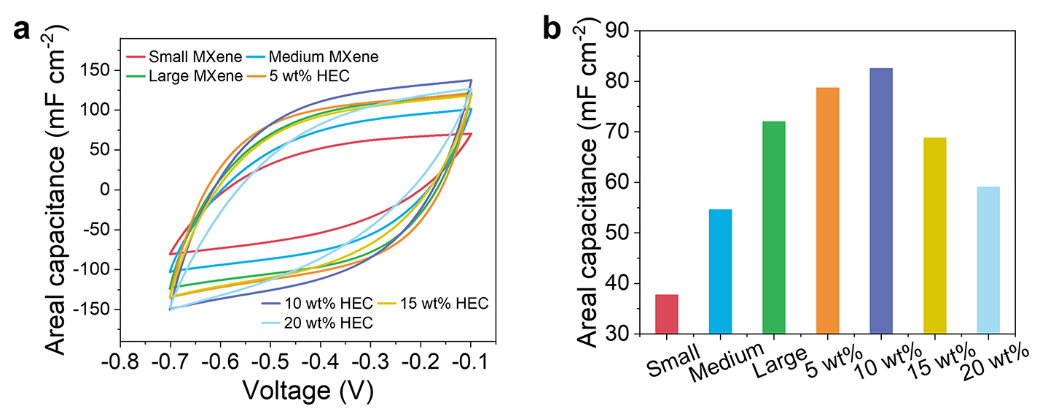


Fig. S26 Electrochemical performance of various MXene electrodes in artificial sweat. (**a**) Cyclic voltammetry curves with scan rates of 20 mV/s. (**b**) Corresponding capacitances


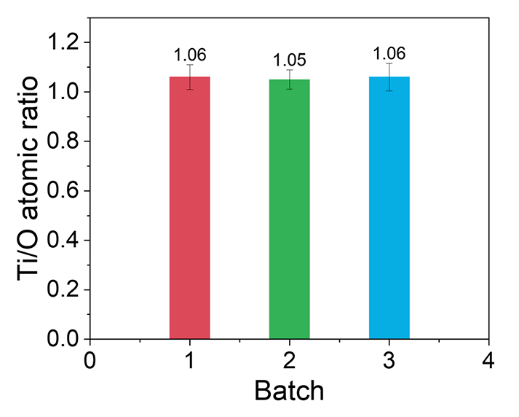


**Fig. S27** Ti/O atomic ratios in 10 wt% HEC/MXene films determined by XPS across different batches. Data are presented as mean ± SD (*n* = 4).


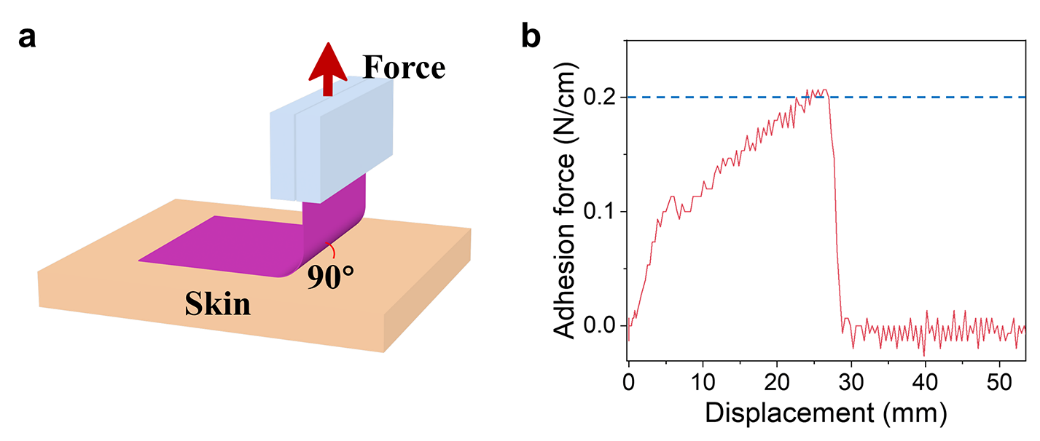


Fig. S28 Adhesiveness of HEC/MXene epidermal electrodes. (**a**) The schematic of the 90° peel test. (**b**) Adhesion forces of HEC/MXene epidermal electrodes on skin


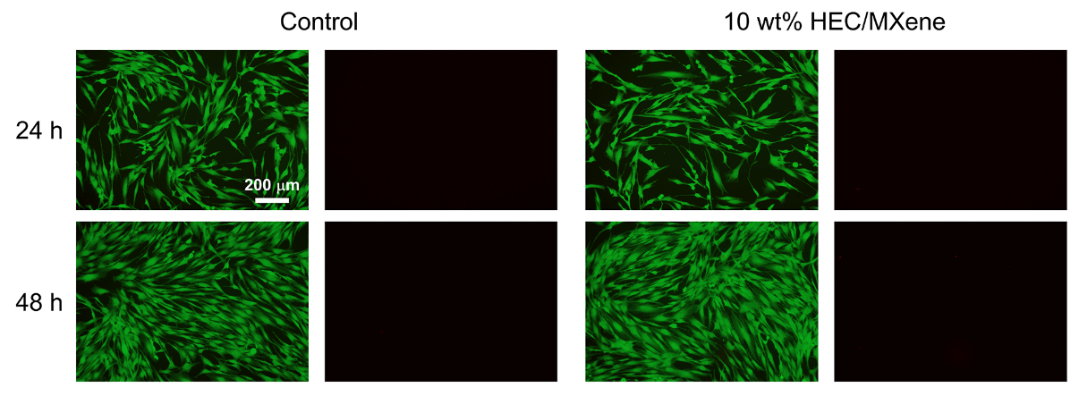


Fig. S29 Fluorescence microscopy images (green: viable cells, red: dead cells) of live/dead human skin fibroblast cells in the control and 10 wt% HEC/MXene film groups after 24 and 48 h immersion


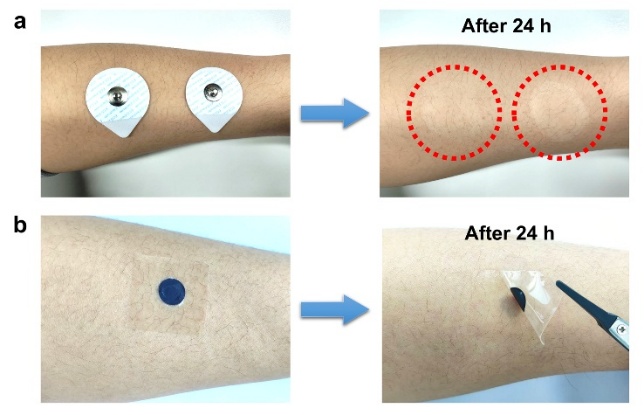


Fig. S30 Irritation comparison between commercial Ag/AgCl gel electrodes and 10 wt% HEC/MXene epidermal electrodes. Photographic images of (**a**) commercial Ag/AgCl gel electrodes and (**b**) 10 wt% HEC/MXene epidermal electrodes worn on human skin for 24 h


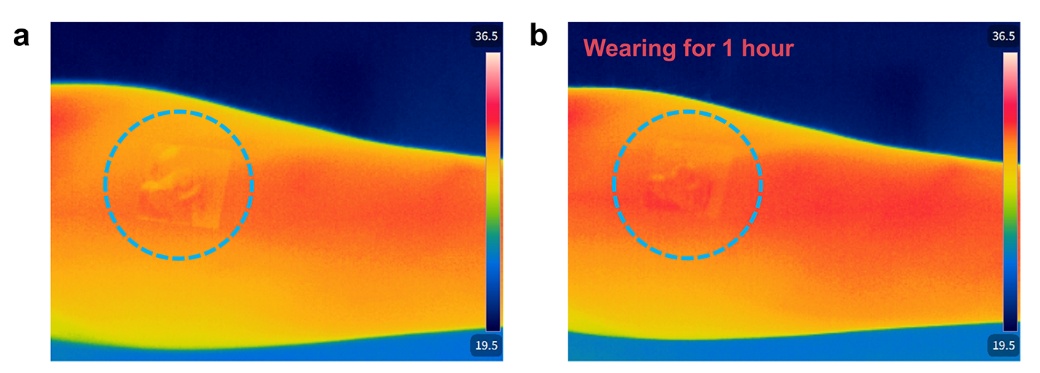


Fig. S31 The infrared images of HEC/MXene epidermal electrode on skin. (**a**) The start of wear. (**b**) After wearing for 1 hour


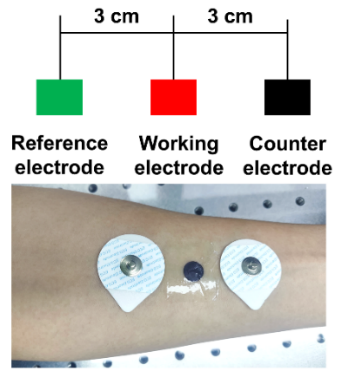


Fig. S32 Electrode-skin interfacial impedance measurement


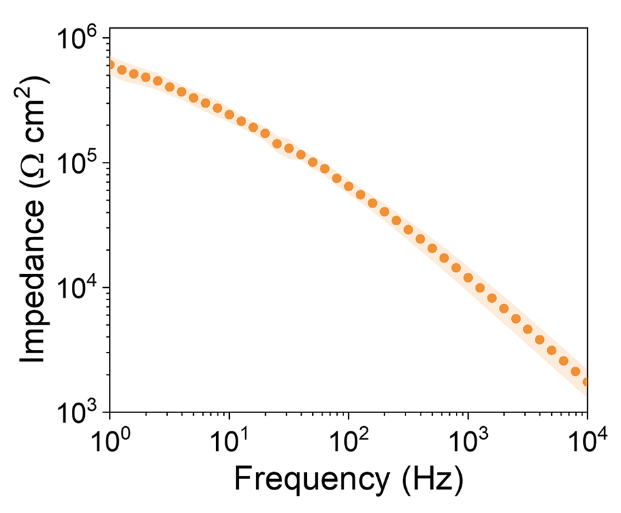


**Fig. S33** Electrode-skin interfacial impedance of small MXene epidermal electrodes Data are presented as mean with shaded regions corresponding to SD (*n* = 5). Data recorded from *N* = 1 subject


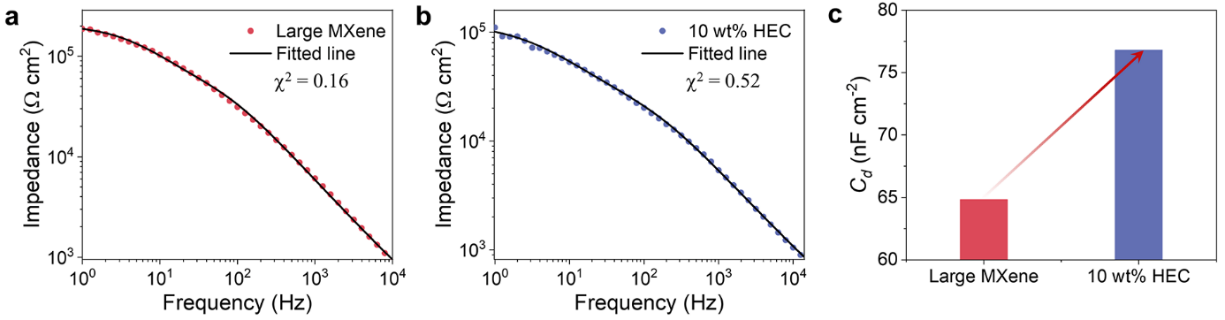


Fig. S34 Electrode-skin interfacial impedance characteristics. Bode magnitude plots for (**a**) large MXene and (**b**) 10 wt% HEC/MXene. The black lines in each plot represent fitted lines. (**c**) Double-layer capacitance as determined by fitting the impedance data of large MXene and 10 wt% HEC/MXene


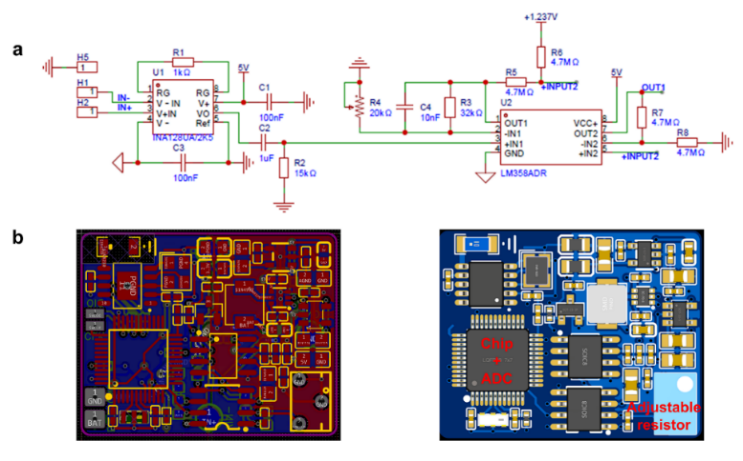


Fig. S35 Data acquisition device of portable EMG monitoring system. (**a**) Data acquisition circuit. (**b**) PCB diagram

The component housed within the blue enclosure is an adjustable resistor, specifically model 3266W.


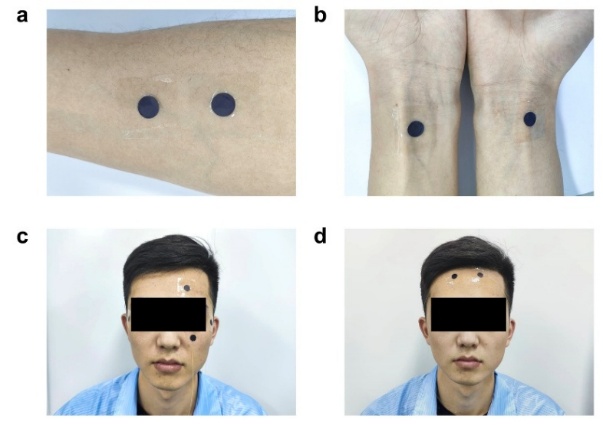


Fig. S36 Photographic images of the experimental setup during electrophysiological monitoring. (**a**) EMG. (**b**) ECG. (**c**) EOG. (**d**) EEG


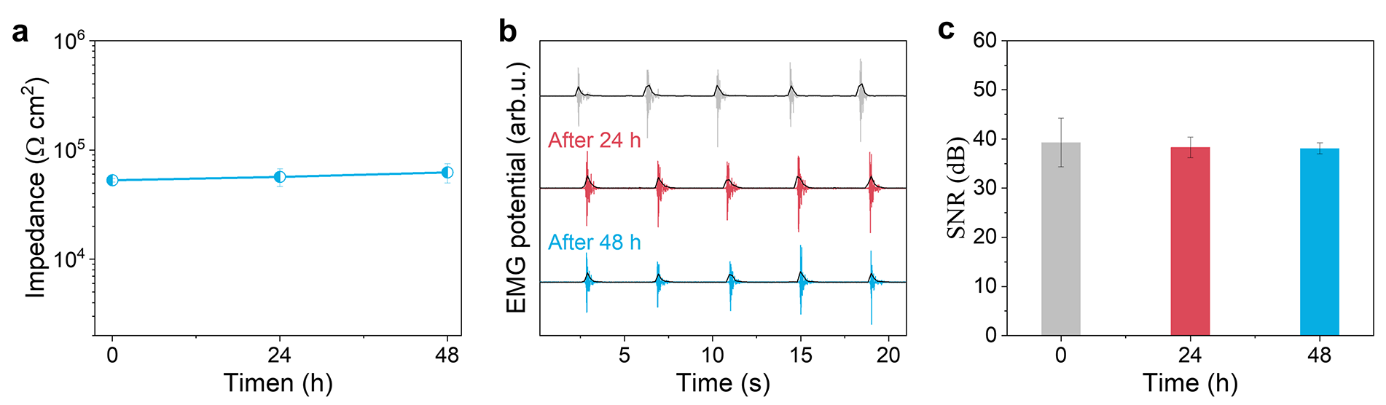


Fig. S37 Long-term EMG monitoring performance of the HEC/MXene epidermal electrodes. (**a**) Evolution of the electrode-skin interfacial impedance over 48 hours. Data are presented as mean ± SD (*n* = 3). (**b**) Recorded EMG signals during the 48-hour monitoring period. (**c**) Corresponding signal-to-noise ratio (SNR) variation over time. Data are presented as mean ± SD (*n* = 8 trials)


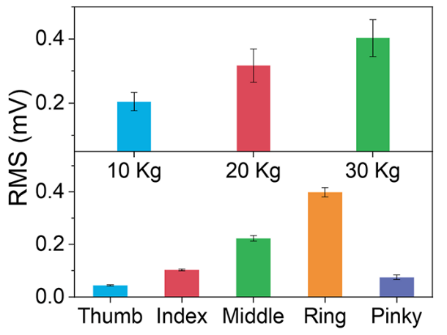


**Fig. S38** RMS values of EMG signal amplitudes. The top corresponds to different grip strengths, and the bottom corresponds to different hand gestures. Data are presented as mean ± SD (*n* = 8 trials)

**
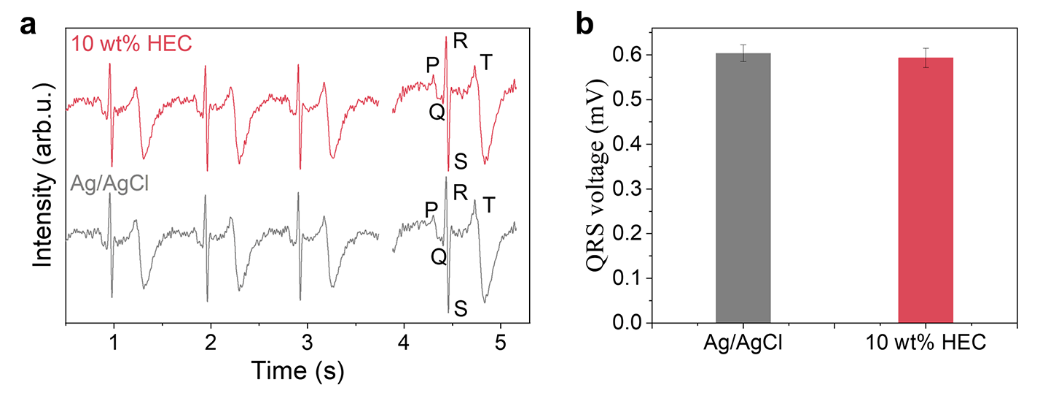
**

Fig. S39 ECG signals detected by commercial Ag/AgCl gel electrodes and HEC/MXene epidermal electrodes. (**a**) Complexes of ECG signals. (**b**) RQS voltage. Data are presented as mean ± SD (*n* = 8 trials)


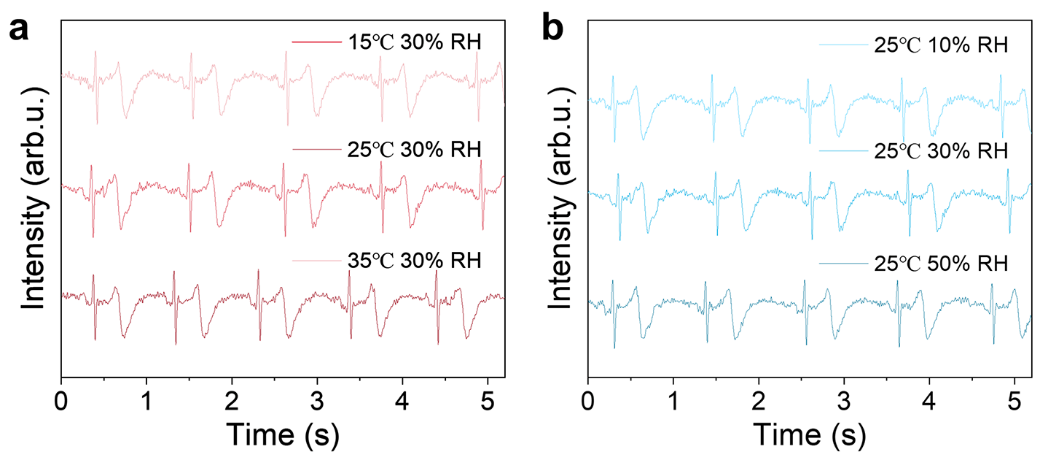


Fig. S40 ECG signals detected by HEC/MXene epidermal electrodes under different temperatures and relative humidity (RH) conditions. (**a**) Different temperatures conditions. (**b**) Different RH conditions

**
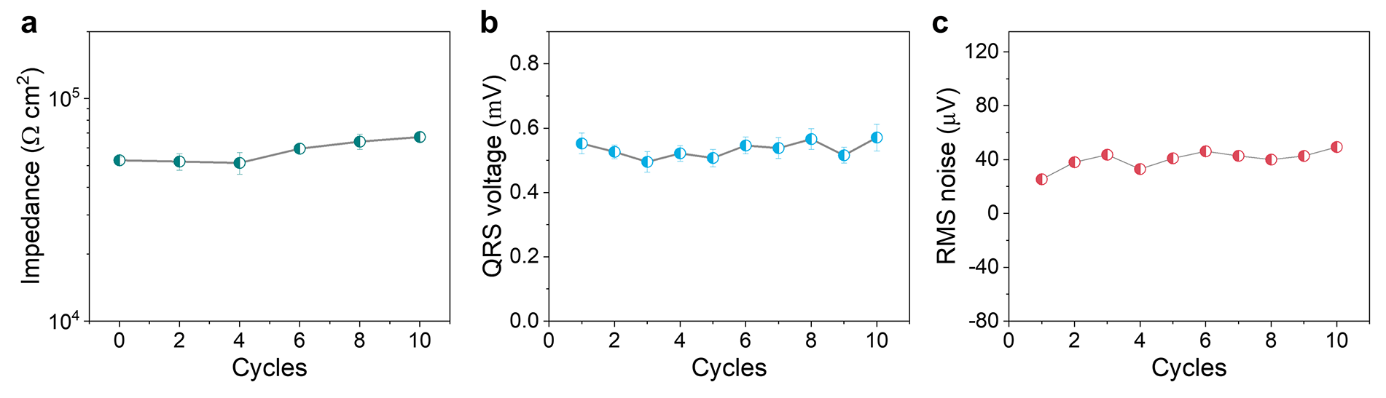
**

Fig. S41 Multi-cycle ECG monitoring performance of the HEC/MXene epidermal electrodes. (**a**) Evolution of the electrode-skin interfacial impedance over cycles. Data are presented as mean ± SD (*n* = 3). (**b**) Variation of QRS voltage observed during multi-cycle ECG monitoring. Data are presented as mean ± SD (*n* = 8 trials). (**c**) Variation of RMS noise observed during multi-cycle ECG monitoring with HEC/MXene epidermal electrodes


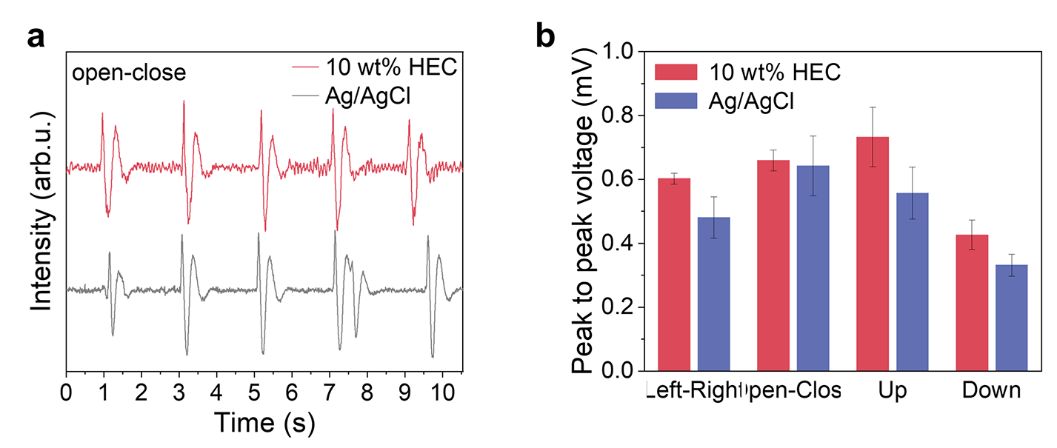


Fig. S42 EOG signals for commercial Ag/AgCl gel electrodes and 10 wt% HEC/MXene epidermal electrodes. (**a**) EOG signals during open-close eye movements. (**b**) Peak-to-peak voltages during varied eye movements. Data are presented as mean ± SD (*n* = 8 trials)


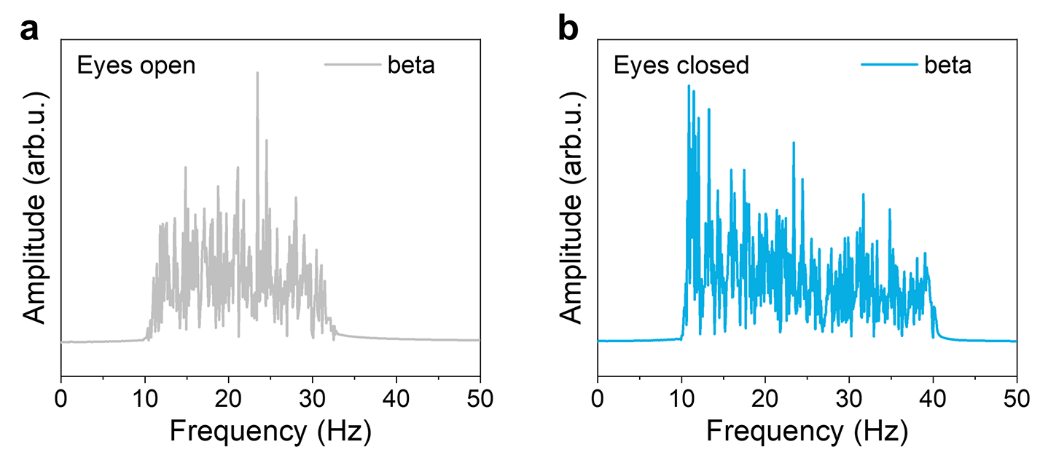


Fig. S43 Extracted beta wave components during different states. (**a**) Eyes open states. (**b**) Eyes closed states

Table S1 Statistical data of various Ti_3_C_2_T*_x_* MXene size distributions

| **Distributions (μm)** | | **Medium Ti_3_C_2_T*_x_* MXene** | | **Large Ti_3_C_2_T*_x_* MXene** | |
| --- | --- | --- | --- | --- | --- |
| Bin range | Bin center | *x* counts | *y* counts | *x* counts | *y* counts |
| 0-1 | 0.5 | 1 | 10 | \ | \ |
| 1-2 | 1.5 | 47 | 135 | \ | \ |
| 2-3 | 2.5 | 63 | 114 | 6 | 53 |
| 3-4 | 3.5 | 83 | 57 | 38 | 74 |
| 4-5 | 4.5 | 53 | 26 | 28 | 50 |
| 5-6 | 5.5 | 33 | 14 | 46 | 29 |
| 6-7 | 6.5 | 24 | 5 | 37 | 16 |
| 7-8 | 7.5 | 19 | 3 | 21 | 2 |
| 8-9 | 8.5 | 14 | 1 | 11 | 6 |
| 9-10 | 9.5 | 10 | 10 | 15 | 1 |
| 10-11 | 10.5 | 7 | \ | 10 | \ |
| 11-12 | 11.5 | 3 | \ | 7 | \ |
| 12-13 | 12.5 | 0 | \ | 7 | \ |
| 13-14 | 13.5 | 3 | \ | 3 | \ |
| 14-15 | 14.5 | 2 | \ | 2 | \ |
| 15-16 | 15.5 | 3 | \ | 1 | \ |
| Total counts | | 365 | 365 | 232 | 232 |

Table S2 XPS fitting results for various Ti_3_C_2_T*_x_* MXene

| **Region** | **Binding energy (eV)** | | | **Assigned to** |
| --- | --- | --- | --- | --- |
|  | small Ti_3_C_2_T*_x_* | Medium Ti_3_C_2_T*_x_* | Large Ti_3_C_2_T*_x_* |  |
| Ti 2p_3/2_ (2p1_/2_) | 454.7 (460.7) | 454.7 (460.7) | 454.7 (460.7) | Ti (I, II or IV) |
|  | 455.3 (461.3) | 455.3 (461.3) | 455.3 (461.3) | Ti^2+^ (I, II, or IV) |
|  | 456.2 (462.2) | 456.2 (462.2) | 456.2 (462.2) | Ti^3+^ (I, II, or IV) |
|  | 459.2 (464.5) | 459.3 (464.6) | 459.2 (464.5) | TiO_2_ |
| C 1s | 281.8 | 281.8 | 281.7 | C-Ti-T*_x_* (I, II,III, or IV) |
|  | 284.8 | 284.8 | 284.8 | C-C |
| O 1s | 529.6 | 529.4 | 529.5 | Ti-O |
|  | 530.9 | 530.3 | 530.3 | C-Ti-O*_x_* |
|  | 532.6 | 532.6 | 532.6 | C-Ti-(OH)*_x_* |
| F 1s | 684.9 | 684.9 | 685.1 | C-Ti-F*_x_* |

Table S3 Fitting parameters of Ti_3_C_2_T*_x_* MXene nanosheets with various flake sizes

| **Size** | **Time constant (**$\boldsymbol{\tau}$**)** | $\boldsymbol{A}_{\boldsymbol{unre}}$ | $\boldsymbol{A}_{\boldsymbol{re}}$ | $\frac{\boldsymbol{A}_{\boldsymbol{re}}}{\boldsymbol{A}_{\boldsymbol{re}}\boldsymbol{+}\boldsymbol{A}_{\boldsymbol{unre}}}$ |
| --- | --- | --- | --- | --- |
| small | 5.5 | 0.19 | 0.80 | 0.81 |
| medium | 6.9 | 0.19 | 0.81 | 0.81 |
| large | 7.5 | 0.17 | 0.83 | 0.83 |

*$\tau$ is the time constant (days), $A_{unre}$ is the unreactive/stable MXene nanosheets, and $A_{re}$ is the reactive/unstable MXene nanosheets.

Table S4 The thickness of HEC/MXene films with various HEC loadings

| **HEC content (wt%)** | **Thickness (μm)** |
| --- | --- |
| 0 | 8.0 |
| 5 | 8.3 |
| 10 | 8.5 |
| 15 | 9.0 |
| 20 | 9.6 |

Table S5 XPS fitting results for large Ti_3_C_2_T*_x_* MXene with various HEC loadings

| **Region** | **Binding energy (eV)** | | | | |
| --- | --- | --- | --- | --- | --- |
|  | Large Ti_3_C_2_T*_x_* | 5 wt% HEC | 10 wt% HEC | 15 wt% HEC | 20 wt% HEC |
| Ti 2p_3/2_ (2p1_/2_) | 454.69 (460.69) | 454.85 (460.85) | 454.87 (460.87) | 454.88 (460.88) | 454.89 (460.89) |
|  | 455.32 (461.32) | 455.49 (461.49) | 455.52 (461.52) | 455.53 (461.53) | 455.54 (461.54) |
|  | 456.15 (462.15) | 456.34 (462.34) | 456.38 (462.38) | 456.37 (462.37) | 456.38 (462.38) |
|  | 459.21 (464.51) | 459.3 (464.6) | 459.34 (464.64) | 459.27 (464.57) | 459.32 (464.62) |

Table S6 Frequency ranges of various electrophysiological signals

| **Electrophysiological signals** | **Frequency (Hz)** |
| --- | --- |
| EEG | 0.5-150 |
| ECG | 0.01-250 |
| EMG | 15-500 |
| EOG | 0.1-50 |

Table S7 Reported interfacial impedance values of commercial Ag/AgCl gel electrodes at 10 Hz

| **Source** | **Impedance at 10 Hz (kΩ)** | **Electrode area (cm^2^)** | **Normalized impedance at 10 Hz (kΩ cm^2^)** |
| --- | --- | --- | --- |
| **This work** | 217 | 2.01 | 436 |
| [S8] | ≈300 | 10.5 | 3150 |
| [S9] | ≈240 | 7.07 | 1697 |
| [S10] | ≈500 | 2.1 | 1050 |
| [S11] | \ | \ | 460 |
| [S12] | \ | \ | 252 |

Table S8 Comparison of electrode-skin interface impedance at 10 Hz for different electrodes without skin abrasion

| **Electrode material** | | **Applications** | **Normalized impedance at 10 Hz (kΩ cm^2^)** | **Electrode area (cm^2^)** | **Conductivity （S/cm）** | **Reference** |
| --- | --- | --- | --- | --- | --- | --- |
| PEDOT:PSS/GO/PEGDMA | | EMG, EEG | 43 | 0.785 | 2060 | [S13] |
| MXene/HEC | | EMG, ECG, EOG, EEG | 53 | 0.785 | 1081 | **This work** |
| Epitaxial Graphene | | EEG | 68 | 1 | \ | [S14] |
| MXene/MOF | | ECG, EMG, EOG | 80 | 1 | \ | [S15] |
| PEDOT:PSS/WPU/D-sorbitol | | ECG, EMG, EEG | 82 | \ | 380 | [S16] |
| PEDOT:PSS/PVA/TA | | ECG, EMG | 100 | \ | 122 | [S17] |
| AgNWs/TPU | | ECG | 106 | 1.767 | 4800 | [S18] |
| LIG/PET | | ECG | 240 | 4 | \ | [S19] |
| CNT film | | ECG | 470 | 0.785 | \ | [S20] |
| MXene/WPU | | ECG, EMG, EEG | 480 | 4 | \ | [S21] |
| AgNWs/PU NFs | ECG | 500 | 1.0 | 9190 | [S22] |  |
| AgNW/HPAN/PU | EMG, ECG | 1400 | 2.0 | 1.5 × 10^-4^ | [S23] |  |
| SWCNT/rGO | ECG | 1570 | 0.785 | \ | [S24] |  |
| MXene/CNF/PCE | ECG, EMG | 3180 | 7.07 | \ | [S9] |  |

Table S9 Signal-to-noise ratios (SNRs) of EMG signals acquired by HEC/MXene electrodes and Ag/AgCl gel electrodes across different subjects

| **Subject** | **Age** | **Gender** | **EMG SNR (dB)** | |
| --- | --- | --- | --- | --- |
|  |  |  | **HEC/MXene electrodes** | **Ag/AgCl gel electrodes** |
| #1 | 28 | Male | 39 ± 5 | 16 ± 2 |
| #2 | 25 | Male | 40 ± 1 | 21 ± 3 |
| #3 | 24 | Female | 34 ± 2 | 15 ± 2 |

**Supplementary References**

1. J.G. Webster, A.J. Nimunkar. Medical instrumentation: application and design. 5th edn. (Wiley, Hoboken, N.J, 2020), pp. 268-320.
2. C. Backes, R.J. Smith, N. McEvoy, N.C. Berner, D. McCloskey et al., Edge and confinement effects allow *in situ* measurement of size and thickness of liquid-exfoliated nanosheets. Nat. Commun. **5**, 4576 (2014). <https://doi.org/10.1038/ncomms5576>
3. C.J. Zhang, S. Pinilla, N. McEvoy, C.P. Cullen, B. Anasori et al., Oxidation stability of colloidal two-dimensional titanium carbides (MXenes). Chem. Mater. **29**(11), 4848–4856 (2017). <https://doi.org/10.1021/acs.chemmater.7b00745>
4. D. Hanlon, C. Backes, E. Doherty, C.S. Cucinotta, N.C. Berner et al., Liquid exfoliation of solvent-stabilized few-layer black phosphorus for applications beyond electronics. Nat. Commun. **6**, 8563 (2015). <https://doi.org/10.1038/ncomms9563>
5. Q. Zhang, R. Fan, W. Cheng, P. Ji, J. Sheng et al., Synthesis of large-area MXenes with high yields through power-focused delamination utilizing *Vortex* kinetic energy. Adv. Sci. **9**(28), 2202748 (2022). <https://doi.org/10.1002/advs.202202748>
6. X. Huang, P. Wu, A facile, high-yield, and freeze-and-thaw-assisted approach to fabricate MXene with plentiful wrinkles and its application in on-chip micro-supercapacitors. Adv. Funct. Mater. **30**(12), 1910048 (2020). <https://doi.org/10.1002/adfm.201910048>
7. A. Sarycheva, Y. Gogotsi, Raman spectroscopy analysis of the structure and surface chemistry of Ti_3_C_2_T*_x_* MXene. Chem. Mater. **32**(8), 3480–3488 (2020). <https://doi.org/10.1021/acs.chemmater.0c00359>
8. Z. Zhang, J. Yang, H. Wang, C. Wang, Y. Gu et al., A 10-micrometer-thick nanomesh-reinforced gas-permeable hydrogel skin sensor for long-term electrophysiological monitoring. Sci. Adv. **10**(2), eadj5389 (2024). <https://doi.org/10.1126/sciadv.adj5389>
9. S. Lee, D.H. Ho, J. Jekal, S.Y. Cho, Y.J. Choi et al., Fabric-based *Lamina* emergent MXene-based electrode for electrophysiological monitoring. Nat. Commun. **15**(1), 5974 (2024). <https://doi.org/10.1038/s41467-024-49939-x>
10. S. Ji, C. Wan, T. Wang, Q. Li, G. Chen et al., Water-resistant conformal hybrid electrodes for aquatic endurable electrocardiographic monitoring. Adv. Mater. **32**(26), e2001496 (2020). <https://doi.org/10.1002/adma.202001496>
11. Q. Wang, Y. Li, Y. Lin, Y. Sun, C. Bai et al., A generic strategy to create mechanically interlocked nanocomposite/hydrogel hybrid electrodes for epidermal electronics. Nanomicro Lett. **16**(1), 87 (2024). <https://doi.org/10.1007/s40820-023-01314-z>
12. M. Asaduzzaman, O. Faruk, A.A. Samad, H. Kim, M.S. Reza et al., A MOFs-derived hydroxyl-functionalized hybrid nanoporous carbon incorporated laser-scribed graphene-based multimodal skin patch for perspiration analysis and electrocardiogram monitoring. Adv. Funct. Mater. **34**(40), 2405651 (2024). <https://doi.org/10.1002/adfm.202405651>
13. X. Du, L. Yang, X. Shi, C. Ye, Y. Wang et al., Ultrathin bioelectrode array with improved electrochemical performance for electrophysiological sensing and modulation. ACS Nano **18**(51), 34971–34985 (2024). <https://doi.org/10.1021/acsnano.4c13325>
14. S.N. Faisal, M. Amjadipour, K. Izzo, J.A. Singer, A. Bendavid et al., Non-invasive on-skin sensors for brain machine interfaces with epitaxial graphene. J. Neural Eng. **18**(6). 066035 (2021). <https://doi.org/10.1088/1741-2552/ac4085>
15. X. Lin, D. Song, T. Shao, T. Xue, W. Hu et al., A multifunctional biosensor *via* MXene assisted by conductive metal–organic framework for healthcare monitoring. Adv. Funct. Mater. **34**(11), 2311637 (2024). <https://doi.org/10.1002/adfm.202311637>
16. L. Zhang, K.S. Kumar, H. He, C.J. Cai, X. He et al., Fully organic compliant dry electrodes self-adhesive to skin for long-term motion-robust epidermal biopotential monitoring. Nat. Commun. **11**(1), 4683 (2020). <https://doi.org/10.1038/s41467-020-18503-8>
17. J. Cao, X. Yang, J. Rao, A. Mitriashkin, X. Fan et al., Stretchable and self-adhesive PEDOT: PSS blend with high sweat tolerance as conformal biopotential dry electrodes. ACS Appl. Mater. Interfaces **14**(34), 39159–39171 (2022). <https://doi.org/10.1021/acsami.2c11921>
18. Y.J. Fan, P.T. Yu, F. Liang, X. Li, H.Y. Li et al., Highly conductive, stretchable, and breathable epidermal electrode based on hierarchically interactive nano-network. Nanoscale **12**(30), 16053–16062 (2020). <https://doi.org/10.1039/d0nr03189e>
19. G. Murastov, E. Bogatova, K. Brazovskiy, I. Amin, A. Lipovka et al., Flexible and water-stable graphene-based electrodes for long-term use in bioelectronics. Biosens. Bioelectron. **166**, 112426 (2020). <https://doi.org/10.1016/j.bios.2020.112426>
20. B.M. Li, O. Yildiz, A.C. Mills, T.J. Flewwellin, P.D. Bradford et al., Iron-on carbon nanotube (CNT) thin films for biosensing E-Textile applications. Carbon **168**, 673–683 (2020). <https://doi.org/10.1016/j.carbon.2020.06.057>
21. Y. Hao, Q. Yan, H. Liu, X. He, P. Zhang et al., A stretchable, breathable, and self-adhesive electronic skin with multimodal sensing capabilities for human-centered healthcare. Adv. Funct. Mater. **33**(44), 2303881 (2023). <https://doi.org/10.1002/adfm.202303881>
22. Z. Jiang, M.O.G. Nayeem, K. Fukuda, S. Ding, H. Jin et al., Highly stretchable metallic nanowire networks reinforced by the underlying randomly distributed elastic polymer nanofibers *via* interfacial adhesion improvement. Adv. Mater. **31**(37), 1903446 (2019). <https://doi.org/10.1002/adma.201903446>
23. X. Yang, S. Wang, M. Liu, L. Li, Y. Zhao et al., All-nanofiber-based Janus epidermal electrode with directional sweat permeability for artifact-free biopotential monitoring. Small **18**(12), 2106477 (2022). <https://doi.org/10.1002/smll.202106477>
24. A.V. Kuksin, A.S. Morozova, Y.O. Vasilevskaya, E.M. Eganova, K.D. Popovich et al., Dry electrodes based on carbon nanotubes-graphene hybrid nanostructures for long-term ECG monitoring. Diam. Relat. Mater. **159**, 112895 (2025). <https://doi.org/10.1016/j.diamond.2025.112895>
